# Supplementary material for: Neighbourhood prevalence-to-notification ratios for adult bacteriologically-confirmed tuberculosis reveals hotspots of underdiagnosis in Blantyre, Malawi
Source: PLoS One. 2022 May 23;17(5):e0268749. doi: 10.1371/journal.pone.0268749 (PMC9126376; doi:10.1371/journal.pone.0268749)
Supplement: S1 Equation — (PDF) [file pone.0268749.s001.pdf]

S1 Equation.

Let $Y \sim Pois(\mu)$

$$\Pr\,(\mathrm{Y}_{ij} = y_{ij}) = \left(\frac{\mu_{ij}^{y_{ij}}\ exp(-\mu_{ij})}{y_{ij}!}\right)$$

$$\log(\mu_{ij}) = \alpha + \beta_1 x_{1i} + \beta_2 x_{2i} \ldots + \beta_k x_{ki} + \beta_{year2015} year2015_j + \beta_{year2016} year2016_j + \beta_{year2017} year2017_j + \beta_{year2018} year2018_j + \log(Pop_{ij}) + \phi_i$$

$$\alpha \sim Normal(\mu_\alpha = 0, \sigma_\alpha^2 = 10)$$

$$\beta_k \sim Normal(\mu_\beta = 0, \sigma_\beta^2 = 10)$$

$$\phi_i \mid \phi_k, k \neq i, \sim Normal\left(\frac{\sum_{i\sim k}\phi_i}{d_i}, \frac{\sigma_i^2}{d_i}\right)$$

$$\sigma_i \sim HalfCauchy(0,1)$$

The expectation of $\mathrm{Y}_{\mathrm{ij}} = y_{ij}$ given by:

$$E(Y_{ij} = y_{ij}) = \mu_{ij}$$

Where i refers to neighbourhood i for i =1,2,3..72, and *j* indexes year (2015,…2019), W is a 72 by 72 adjacency matrix where entries {i,i} are 0 and the off-diagonal elements are 1 if regions i and k are neighbours and 0 otherwise. d_i is the number of neighbours for neighbourhood$_i$, d_i was fixed to be 4. ϕ_i , α and $\beta_1, \beta_2 \ldots \beta_k$ are unknown regression coefficients that are estimated from the data for the covariates $x_{1i}, x_{2i} \ldots x_{ki}$, and sigma (σ_i) is the standard deviation for the spatial random term ϕ_i. Pop_{ij} is the total population of neighbourhood that is used as the offset. Here $year2015_j$, $year2016_j$, $year2017_j$ and $year2018_j$ are dummy variables which take the value of one for that year and take the value of zero for the other years (the baseline year was 2019).
