# Supplementary material for: Neighbourhood prevalence-to-notification ratios for adult bacteriologically-confirmed tuberculosis reveals hotspots of underdiagnosis in Blantyre, Malawi
Source: PLoS One. 2022 May 23;17(5):e0268749. doi: 10.1371/journal.pone.0268749 (PMC9126376; doi:10.1371/journal.pone.0268749)
Supplement: S2 Equation — (PDF) [file pone.0268749.s002.pdf]

**S2 Equation.**

To define the zero-inflated Poisson model, let $Z \sim Bern(1-p)$ (so $\Pr\{Z=0\}=p$) and independently $W \sim Pois(\mu)$. Then the data are modelled by the zero-inflated variable Y which is defined as

$$Y = ZW$$

$$\Pr(Y_i = y_i) = \begin{cases} (p + (1-p)exp(-\mu_i))\ if\ y_i = 0 \\ \left((1-p)\dfrac{\mu_i^{y_i}\ exp(-\mu_i)}{y_i!}\right) if\ y_i \geq 1 \end{cases}$$

$$logit(p_i) = \vartheta$$

$$\vartheta \sim Normal(\mu = 0, \sigma_\vartheta^2 = 10)$$

$$\log(\mu_i) = \alpha + \beta_1 x_{1i} + \beta_2 x_{2i} \ldots + \beta_k x_{ki} + \log(Pop_i) + \phi_i$$

$$\alpha \sim Normal(\mu_\alpha = 0, \sigma_\alpha^2 = 10)$$

$$\beta_k \sim Normal(\mu_\beta = 0, \sigma_\beta^2 = 10)$$

$$\phi_i \mid \phi_k, k \neq i, \sim Normal\left(\frac{\sum_{i \sim k} \phi_i}{d_i}, \frac{\sigma_i^2}{d_i}\right)$$

$$\sigma_i \sim HalfCauchy(0,1)$$

The expectation of $Y_i = y_i$ given by:

$$E(Y_i = y_i) = (1-p_i)\,\mu_i$$

Where i refers to neighbourhood i for i =1,2,3..72 (note we only have prevalence data for one year, 2019). W is a 72 by 72 adjacency matrix where entries {i,i} are 0 and the off-diagonal elements are 1 if regions i and k are neighbours and 0 otherwise. $d_i$ is the number of neighbours for neighbourhood$_i$, $d_i$ was fixed to be 4. $\phi_i$, $\theta, \alpha$ and $\beta_1, \beta_2 \ldots \beta_k$ are unknown regression coefficients that are estimated from the data for the covariates $x_{1i}, x_{2i} \ldots x_{ki}$, and sigma ($\sigma_i$) is the standard deviation for the spatial random term $\phi_i$. $Pop_i$ is the total population of neighbourhood that is used as the offset.
