# Supplementary material for: Neighbourhood prevalence-to-notification ratios for adult bacteriologically-confirmed tuberculosis reveals hotspots of underdiagnosis in Blantyre, Malawi
Source: PLoS One. 2022 May 23;17(5):e0268749. doi: 10.1371/journal.pone.0268749 (PMC9126376; doi:10.1371/journal.pone.0268749)
Supplement: S3 Equation — (PDF) [file pone.0268749.s003.pdf]

S3 Equation. Model goodness of fit assessment.

We now present a chi-square-statistic goodness of fit statistic for the models as an approximate guidance to the goodness of fit of the models to the data.

Let Y_i be the observed number of cases and $\widehat{\mu_i}$ the model predicated cases in neighbourhood *i* = 1, 2, 3, …72

$$\chi^2 = \sum_i \frac{(Y_i - \hat{\mu}_i)^2}{\hat{\mu}_i}$$

The value of chi-square was calculated separately for TB case notifications and also for prevalent TB cases. We also calculated the approximate degrees of freedom of each as 72 – number of parameters in each model.

The calculated chi-squared statistic of the case notifications was 73.06, while the degree of freedom was 64, giving an approximate p-value of 0.21
Similarly, the calculated chi-squared statistic of the prevalent cases was 56.81, while the degree of freedom was 70, giving an approximate p-value of 0.87.

While, because expectations in the 72 locations are often small, this only provides an approximate measure of goodness of fit, nevertheless taken together with our careful choice of models (e.g. zero-inflated Poisson for the prevalence) and systematic variable selection approach means are confident our models describe the data sufficiently well to justify their application.
