# Supplementary material for: Neighbourhood prevalence-to-notification ratios for adult bacteriologically-confirmed tuberculosis reveals hotspots of underdiagnosis in Blantyre, Malawi
Source: PLoS One. 2022 May 23;17(5):e0268749. doi: 10.1371/journal.pone.0268749 (PMC9126376; doi:10.1371/journal.pone.0268749)
Supplement: S1 Fig — The neighbourhoods were ordered according to prevalence to notification ratio size. Analysis based on post stratified TB prevalence with microbiologically-confirmed TB notifications kept the same as in the primary analysis. The dashed line is the mean prevalence to notification ratio. Crl Credible interval. (PDF) [file pone.0268749.s004.pdf]

S1 Fig. Neighbourhood level TB prevalence to notification rate ratios (with 95% CIs) using final models. The neighbourhoods were ordered according to prevalence to notification ratio size. Analysis based on post stratified TB prevalence with microbiologically-confirmed TB notifications kept the same as in the primary analysis. The dashed line is the mean prevalence to notification ratio. CNR case notification ratio; CrI Credible interval.

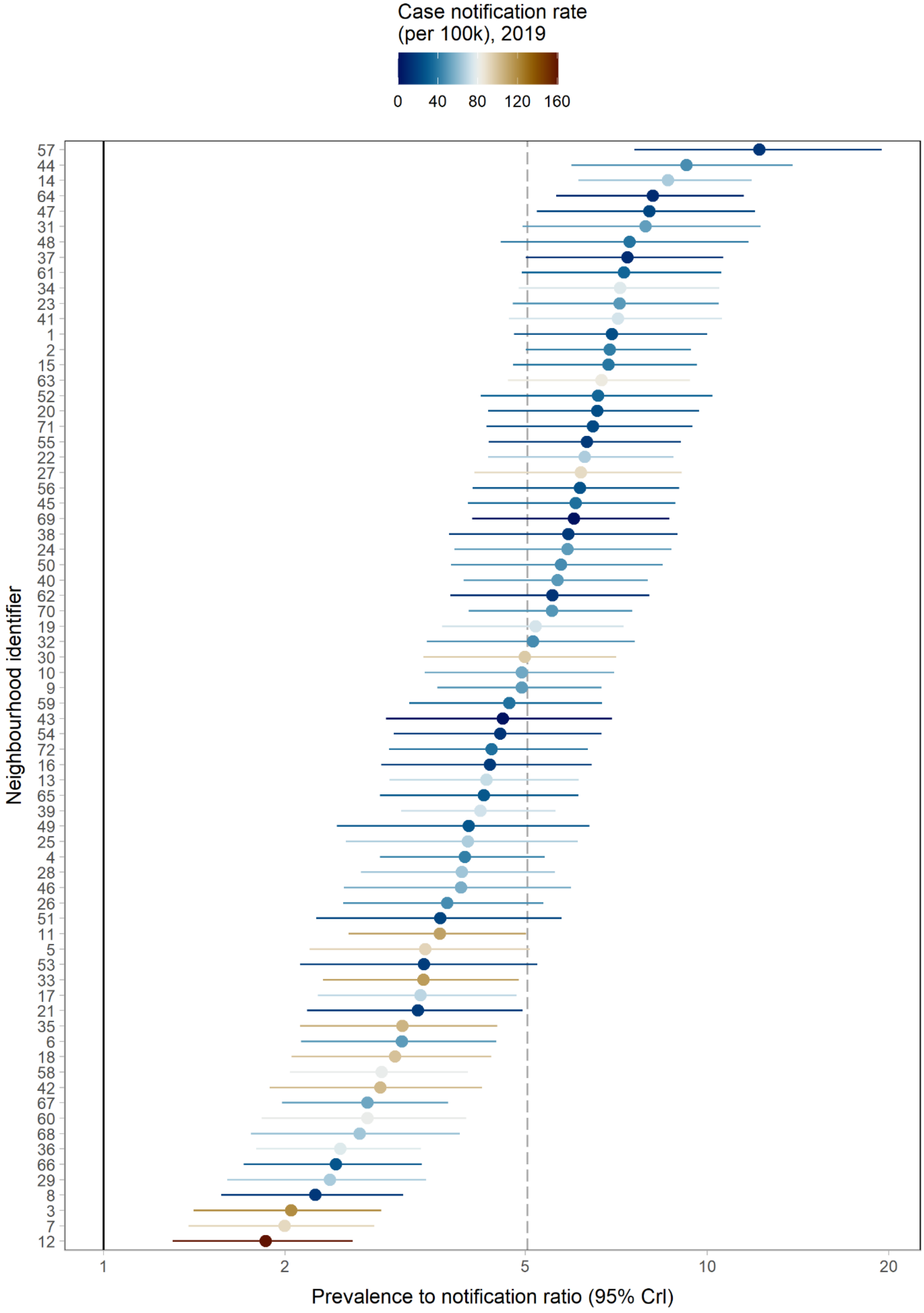

Case notification rate
(per 100k), 2019
0
40
80
120
160
Neighbourhood identifier
57
44
14
64
47
31
48
37
61
34
23
41
1
2
15
63
52
20
71
55
22
27
56
45
69
38
24
50
40
62
70
19
32
30
10
9
59
43
54
72
16
13
65
39
49
25
4
28
46
26
51
11
5
53
33
17
21
35
6
18
58
42
67
60
68
36
66
29
8
3
7
12
1
2
5
10
20
Prevalence to notification ratio (95% CrI)
