# Supplementary material for: Neighbourhood prevalence-to-notification ratios for adult bacteriologically-confirmed tuberculosis reveals hotspots of underdiagnosis in Blantyre, Malawi
Source: PLoS One. 2022 May 23;17(5):e0268749. doi: 10.1371/journal.pone.0268749 (PMC9126376; doi:10.1371/journal.pone.0268749)

S2 Fig. Differences of neighbourhood level TB prevalence to notification rate ratios (P:N rate ratios), the P:N rate ratio based on post stratified prevalence TB subtracted by the P:N ratio based on the primary analysis.

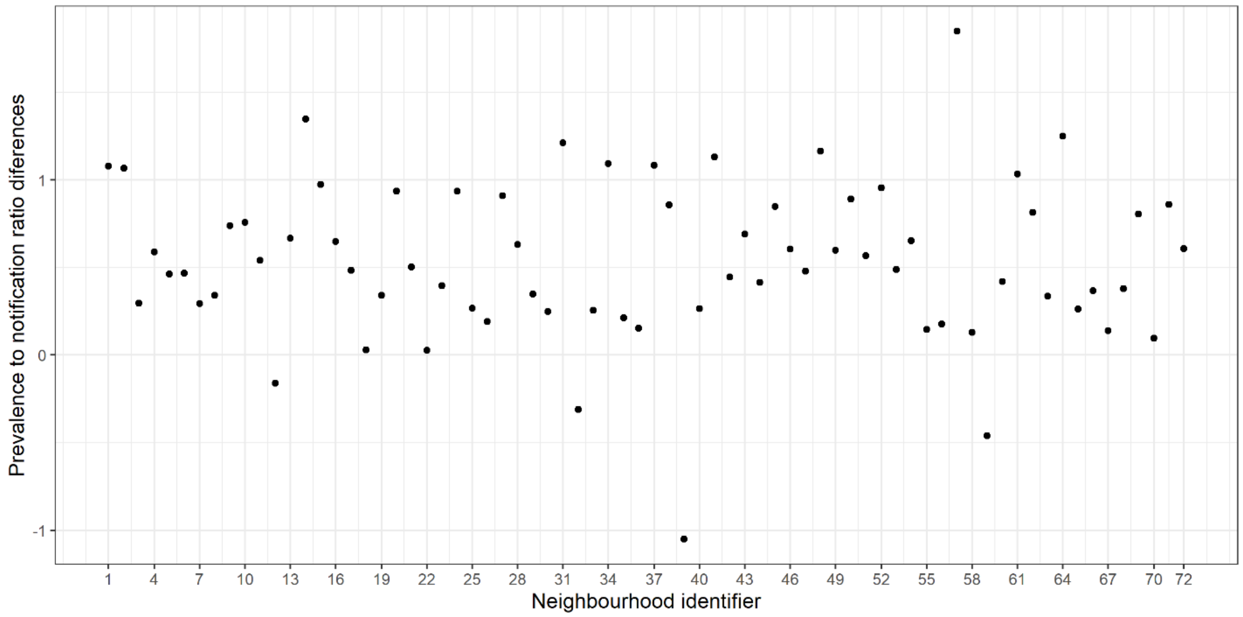

Supplement: S2 Fig — (PDF) [file pone.0268749.s005.pdf]
