# Supplementary material for: Neighbourhood prevalence-to-notification ratios for adult bacteriologically-confirmed tuberculosis reveals hotspots of underdiagnosis in Blantyre, Malawi
Source: PLoS One. 2022 May 23;17(5):e0268749. doi: 10.1371/journal.pone.0268749 (PMC9126376; doi:10.1371/journal.pone.0268749)

S3 Fig. Map of TB prevalence to notification ratios predicted from final models (Inset map of Malawi with Blantyre in red). Analysis based on post stratified TB prevalence and with microbiologically confirmed TB notifications kept the same as in the primary analysis. Models include neighbourhood random effects. Neighbourhoods outlined in blue are in the highest quartile for P:N ratios. Inset map of Malawi with Blantyre District in red.

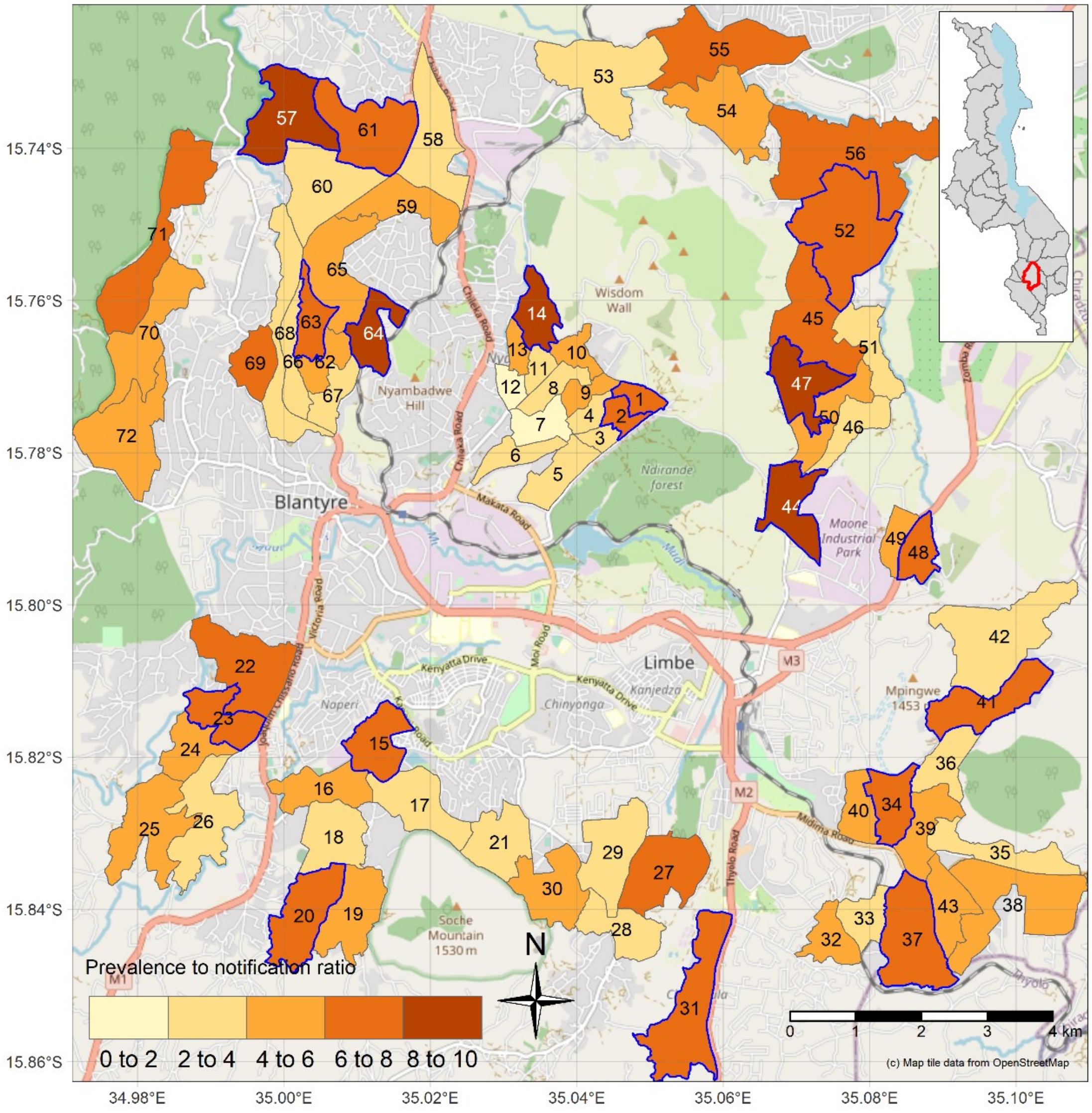

Supplement: S3 Fig — Analysis based on post stratified TB prevalence and with microbiologically-confirmed TB notifications kept the same as in the primary analysis. Models include neighbourhood random effects. Neighbourhoods outlined in blue are in the highest quartile for P:N ratios. Map tile data from OpenStreetMap. (PDF) [file pone.0268749.s006.pdf]
