# Supplementary material for: Neighbourhood prevalence-to-notification ratios for adult bacteriologically-confirmed tuberculosis reveals hotspots of underdiagnosis in Blantyre, Malawi
Source: PLoS One. 2022 May 23;17(5):e0268749. doi: 10.1371/journal.pone.0268749 (PMC9126376; doi:10.1371/journal.pone.0268749)
Supplement: S4 Fig — The neighbourhoods were ordered according to prevalence to notification ratio size. Analysis based on microbiologically-confirmed TB and clinically-diagnosed cases and with TB prevalence kept the same as in the primary analysis. The dashed line is the mean prevalence to notification ratio. Crl Credible interval. (PDF) [file pone.0268749.s007.pdf]

S4 Fig. Neighbourhood level TB prevalence to notification rate ratios (with 95% CIs) using final models. The neighbourhoods were ordered according to prevalence to notification ratio size. Analysis based on microbiologically-confirmed TB and clinically-diagnosed cases and with TB prevalence kept the same as in the primary analysis. The dashed line is the mean prevalence to notification ratio. Crl Credible interval.

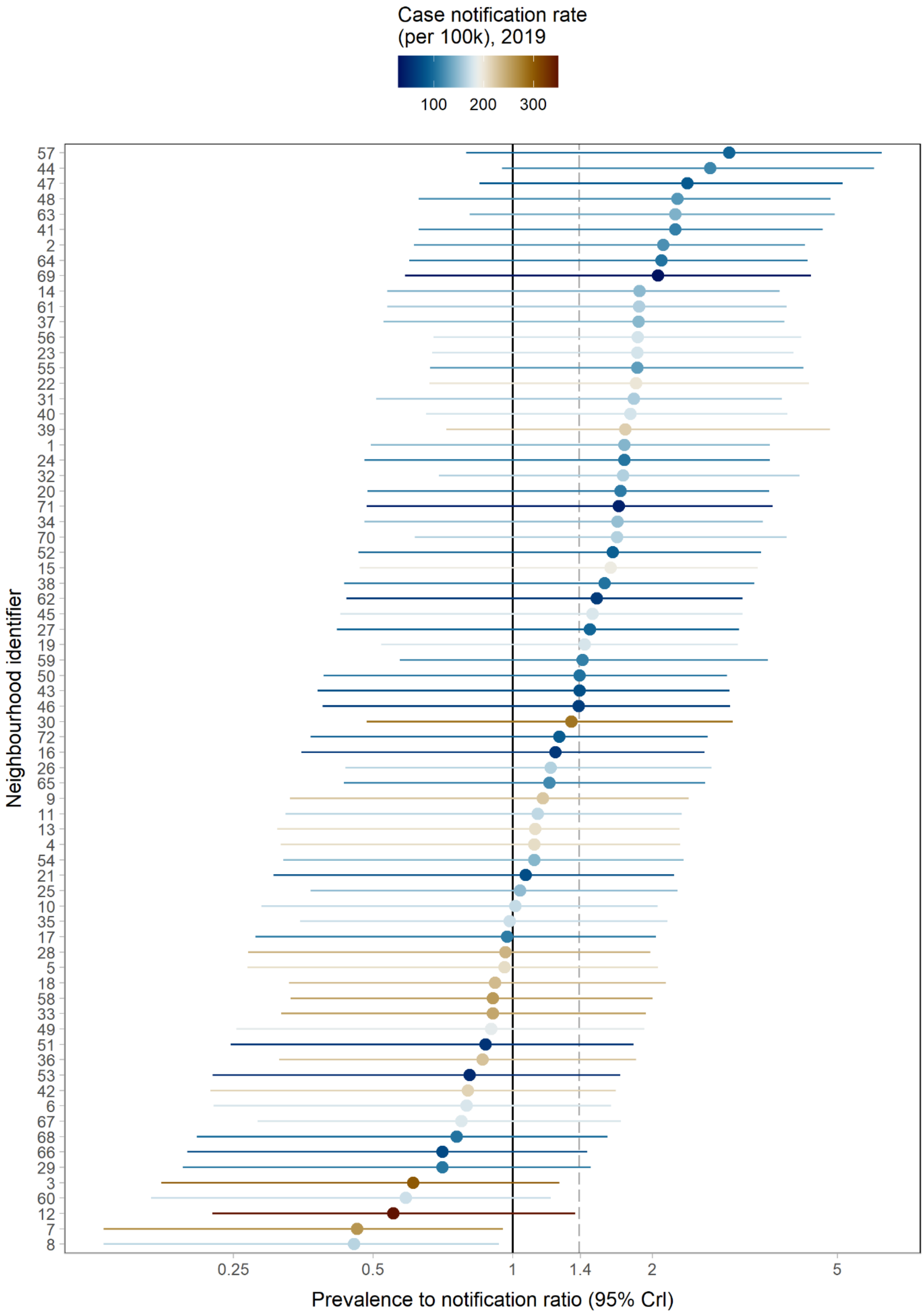

Case notification rate
(per 100k), 2019
100
200
300
Neighbourhood identifier
57
44
47
48
63
41
2
64
69
14
61
37
56
23
55
22
31
40
39
1
24
32
20
71
34
70
52
15
38
62
45
27
19
59
50
43
46
30
72
16
26
65
9
11
13
4
54
21
25
10
35
17
28
5
18
58
33
49
51
36
53
42
6
67
68
66
29
3
60
12
7
8
0.25
0.5
1
1.4
2
5
Prevalence to notification ratio (95% CrI)
