# Supplementary material for: Neighbourhood prevalence-to-notification ratios for adult bacteriologically-confirmed tuberculosis reveals hotspots of underdiagnosis in Blantyre, Malawi
Source: PLoS One. 2022 May 23;17(5):e0268749. doi: 10.1371/journal.pone.0268749 (PMC9126376; doi:10.1371/journal.pone.0268749)

S5 Fig. Map of TB prevalence to notification ratios predicted from final models (Inset map of Malawi with Blantyre in red). Analysis based on microbiologically-confirmed TB and clinically-diagnosed cases and with TB prevalence kept the same as in the primary analysis. Models include neighbourhood random effects. Neighbourhoods outlined in blue are in the highest quartile for P:N ratios.

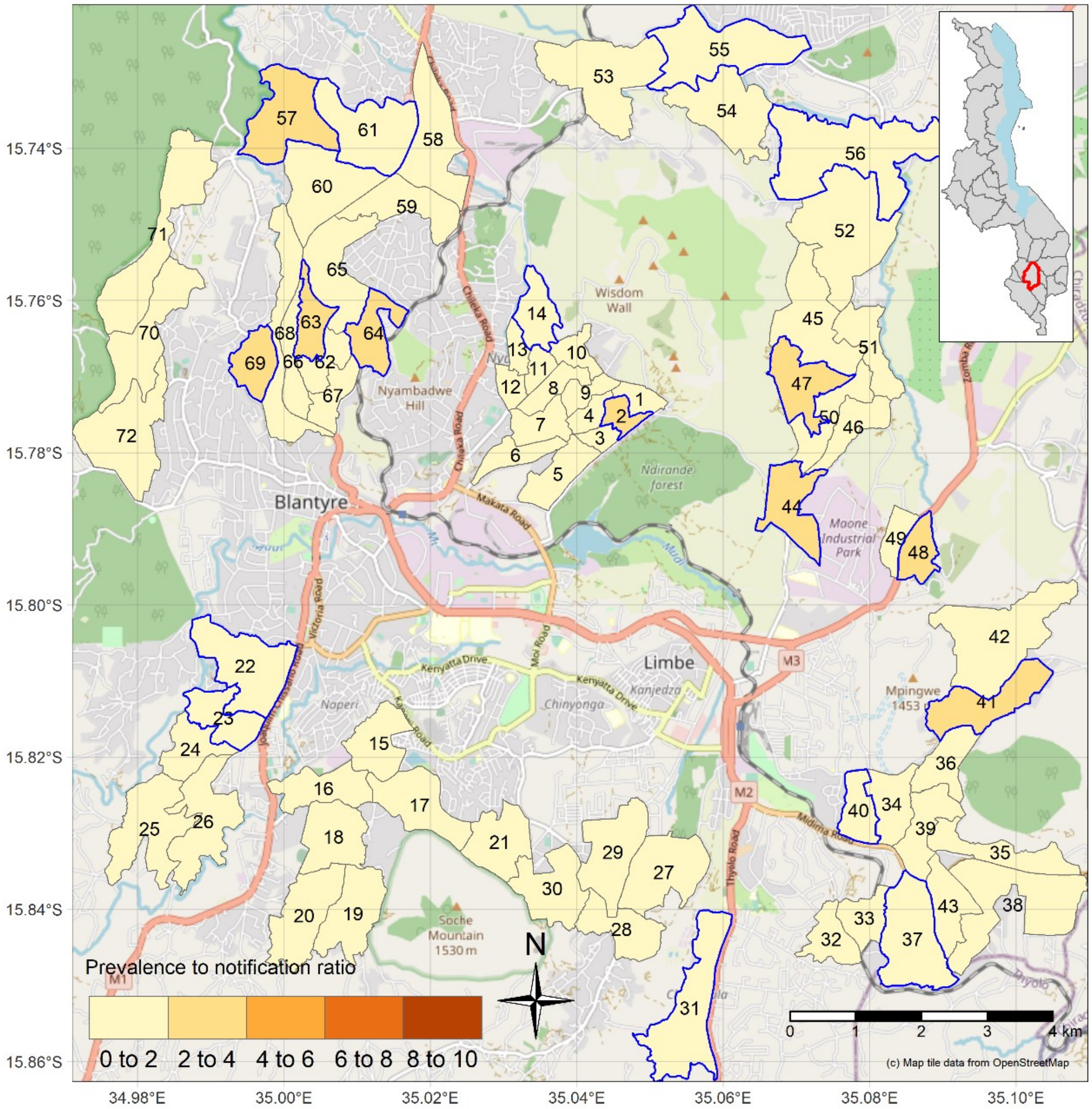

Supplement: S5 Fig — Analysis based on microbiologically-confirmed TB and clinically-diagnosed cases and with TB prevalence kept the same as in the primary analysis. Models include neighbourhood random effects. Neighbourhoods outlined in blue are in the highest quartile for P:N ratios. Map tile data from OpenStreetMap. (PDF) [file pone.0268749.s008.pdf]
