# Supplementary material for: Neighbourhood prevalence-to-notification ratios for adult bacteriologically-confirmed tuberculosis reveals hotspots of underdiagnosis in Blantyre, Malawi
Source: PLoS One. 2022 May 23;17(5):e0268749. doi: 10.1371/journal.pone.0268749 (PMC9126376; doi:10.1371/journal.pone.0268749)

S7 Fig. Observed versus predicted mean prevalence rates (95% CrIs). Analysis based on TB prevalence as in the primary analysis. CrI Credible interval.

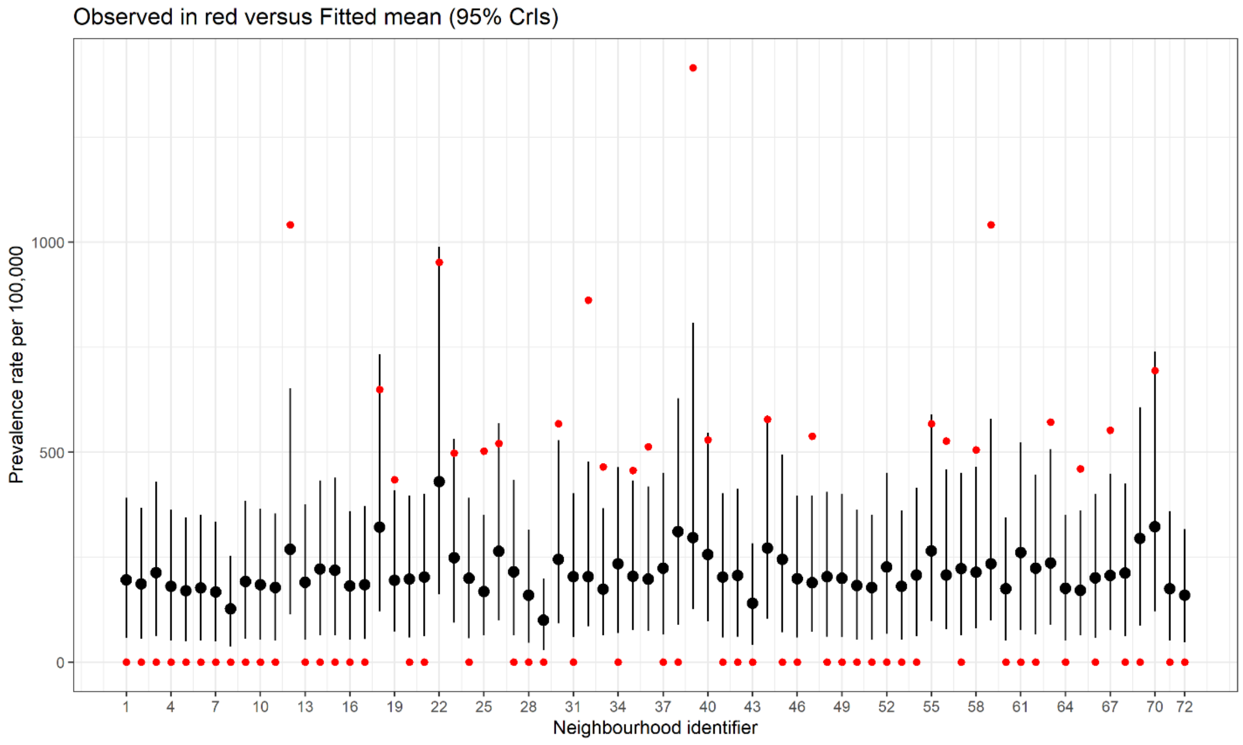

Supplement: S7 Fig — Analysis based on microbiologically-confirmed TB as in the primary analysis. Crl Credible interval. (PDF) [file pone.0268749.s010.pdf]
