# Supplementary material for: Neighbourhood prevalence-to-notification ratios for adult bacteriologically-confirmed tuberculosis reveals hotspots of underdiagnosis in Blantyre, Malawi
Source: PLoS One. 2022 May 23;17(5):e0268749. doi: 10.1371/journal.pone.0268749 (PMC9126376; doi:10.1371/journal.pone.0268749)
Supplement: S1 Table — Coefficients (mean rate ratio) were exponentiated and intercepts were multiplied by 100,000. (PDF) [file pone.0268749.s011.pdf]

S1 Table. Table of all the TB prevalence neighbourhood level models with a random intercept of clinic of treatment registration. Coefficients (mean rate ratio) were exponentiated and intercepts were multiplied by 100,000 (Equation 2).

Model names	Intercept	Percentage of adults (≥15y)	Percentage of household heads that did not complete primary school	Distance to nearest TB clinic (km)	Percentage of HIV prevalence	Percentage of male adults	Random effects SD: cluster	Probability Zi=0 (Refer to Equation 2)
prevalence model 1	218.46 (98.53-463.00)	0.90 (0.72-1.13)	0.99 (0.91-1.08)	0.98 (0.55-1.74)	1.03 (0.91-1.16)	0.92 (0.64-1.33)	0.39 (0.02-1.05)	0.19 (0.01-0.48)
prevalence model 2	210.26 (96.11-449.69)		1.01 (0.93-1.09)	1.08 (0.63-1.86)	1.02 (0.91-1.15)	0.98 (0.70-1.38)	0.38 (0.02-1.04)	0.19 (0.01-0.48)
prevalence model 3	220.73 (101.37-462.95)	0.91 (0.73-1.12)		0.99 (0.55-1.75)	1.02 (0.91-1.14)	0.92 (0.63-1.32)	0.37 (0.01-1.00)	0.19 (0.01-0.48)
prevalence model 4	212.01 (97.42-448.19)			1.09 (0.64-1.85)	1.03 (0.92-1.15)	0.98 (0.71-1.36)	0.36 (0.02-0.99)	0.19 (0.01-0.47)
prevalence model 5	217.90 (116.83-396.19)	0.90 (0.73-1.11)	0.99 (0.91-1.08)		1.03 (0.91-1.15)	0.92 (0.64-1.32)	0.37 (0.02-1.01)	0.19 (0.01-0.47)
prevalence model 6	226.71 (125.32-406.29)		1.01 (0.94-1.09)		1.02 (0.91-1.15)	0.99 (0.73-1.36)	0.36 (0.01-0.97)	0.19 (0.01-0.47)
prevalence model 7	220.83 (119.92-398.84)	0.91 (0.75-1.10)			1.02 (0.91-1.14)	0.92 (0.64-1.31)	0.36 (0.01-0.98)	0.18 (0.01-0.47)
prevalence model 8	230.62 (129.55-405.77)				1.02 (0.92-1.14)	1.00 (0.75-1.34)	0.34 (0.02-0.94)	0.18 (0.01-0.47)
prevalence model 9	223.22 (104.13-461.02)	0.91 (0.72-1.12)	1.00 (0.92-1.08)	0.97 (0.54-1.73)		0.89 (0.64-1.24)	0.37 (0.01-1.02)	0.19 (0.01-0.47)
prevalence model 10	214.86 (102.66-445.87)		1.01 (0.94-1.09)	1.07 (0.62-1.80)		0.95 (0.71-1.28)	0.36 (0.01-0.99)	0.18 (0.01-0.47)
prevalence model 11	226.52 (105.67-462.82)	0.91 (0.74-1.10)		0.97 (0.55-1.71)		0.89 (0.65-1.23)	0.36 (0.02-0.98)	0.18 (0.01-0.47)
prevalence model 12	216.23 (103.84-443.79)			1.08 (0.65-1.80)		0.95 (0.71-1.27)	0.35 (0.01-0.97)	0.18 (0.01-0.46)

Model names	Intercept	Percentage of adults (≥15y)	Percentage of household heads that did not complete primary school	Distance to nearest TB clinic (km)	Percentage of HIV prevalence	Percentage of male adults	Random effects SD: cluster	Probability Zi=0 (Refer to Equation 2)
prevalence model 13	221.57 (120.89-394.66)	0.91 (0.74-1.11)	1.00 (0.92-1.08)			0.89 (0.65-1.23)	0.36 (0.01-0.98)	0.18 (0.01-0.46)
prevalence model 14	229.64 (128.60-402.07)		1.01 (0.95-1.09)			0.96 (0.74-1.26)	0.35 (0.01-0.93)	0.18 (0.01-0.46)
prevalence model 15	225.02 (125.63-394.43)	0.91 (0.75-1.09)				0.89 (0.65-1.22)	0.35 (0.01-0.94)	0.18 (0.01-0.46)
prevalence model 16	233.95 (132.60-407.26)					0.97 (0.75-1.26)	0.33 (0.01-0.91)	0.18 (0.01-0.46)
prevalence model 17	223.37 (103.70-459.25)	0.92 (0.74-1.12)	0.99 (0.91-1.08)	0.97 (0.55-1.71)	1.04 (0.93-1.16)		0.36 (0.01-1.00)	0.19 (0.01-0.47)
prevalence model 18	214.82 (102.48-438.95)		1.01 (0.93-1.08)	1.07 (0.64-1.76)	1.03 (0.92-1.14)		0.35 (0.01-0.98)	0.18 (0.01-0.47)
prevalence model 19	227.46 (108.74-461.39)	0.93 (0.77-1.11)		0.97 (0.55-1.68)	1.03 (0.93-1.14)		0.35 (0.01-0.95)	0.18 (0.01-0.46)
prevalence model 20	216.39 (105.62-431.03)			1.08 (0.66-1.74)	1.03 (0.93-1.13)		0.35 (0.01-0.95)	0.18 (0.01-0.47)
prevalence model 21	220.72 (122.28-392.12)	0.92 (0.76-1.10)	0.99 (0.91-1.07)		1.04 (0.93-1.15)		0.35 (0.01-0.97)	0.18 (0.01-0.46)
prevalence model 22	228.49 (128.32-398.52)		1.01 (0.94-1.08)		1.02 (0.92-1.12)		0.35 (0.01-0.95)	0.18 (0.01-0.46)
prevalence model 23	224.93 (125.81-395.68)	0.93 (0.79-1.09)			1.03 (0.93-1.14)		0.34 (0.01-0.92)	0.18 (0.01-0.46)
prevalence model 24	233.05 (133.04-405.34)				1.02 (0.93-1.12)		0.33 (0.01-0.91)	0.18 (0.01-0.46)
prevalence model 25	236.34 (112.67-479.93)	0.93 (0.77-1.13)	1.00 (0.93-1.08)	0.94 (0.54-1.61)			0.36 (0.01-0.97)	0.19 (0.01-0.47)

Model names	Intercept	Percentage of adults (≥15y)	Percentage of household heads that did not complete primary school	Distance to nearest TB clinic (km)	Percentage of HIV prevalence	Percentage of male adults	Random effects SD: cluster	Probability Zi=0 (Refer to Equation 2)
prevalence model 26	225.87 (111.64-445.27)		1.01 (0.94-1.08)	1.02 (0.63-1.64)			0.35 (0.01-0.95)	0.18 (0.01-0.46)
prevalence model 27	240.36 (117.23-479.19)	0.93 (0.78-1.12)		0.94 (0.54-1.61)			0.34 (0.01-0.94)	0.18 (0.01-0.47)
prevalence model 28	226.74 (112.73-448.23)			1.04 (0.65-1.65)			0.34 (0.01-0.92)	0.18 (0.01-0.46)
prevalence model 29	227.68 (125.87-401.40)	0.94 (0.79-1.12)	1.00 (0.93-1.08)				0.35 (0.01-0.95)	0.18 (0.01-0.46)
prevalence model 30	233.71 (133.43-407.32)		1.01 (0.94-1.08)				0.33 (0.01-0.90)	0.18 (0.01-0.46)
prevalence model 31	232.08 (132.05-404.96)	0.94 (0.80-1.10)					0.33 (0.01-0.90)	0.18 (0.01-0.46)
prevalence model 32	237.63 (136.98-410.81)						0.32 (0.01-0.89)	0.17 (0.01-0.46)
