# Supplementary material for: Neighbourhood prevalence-to-notification ratios for adult bacteriologically-confirmed tuberculosis reveals hotspots of underdiagnosis in Blantyre, Malawi
Source: PLoS One. 2022 May 23;17(5):e0268749. doi: 10.1371/journal.pone.0268749 (PMC9126376; doi:10.1371/journal.pone.0268749)
Supplement: S2 Table — Coefficients (mean rate ratio) were exponentiated and intercepts were multiplied by 100,000. (PDF) [file pone.0268749.s012.pdf]

S2 Table. Table of all the TB notified neighbourhood level models with a random intercept of clinic of treatment registration. Coefficients (mean rate ratio) were exponentiated and intercepts were multiplied by 100,000 (Equation 1).

Model names	Intercept	Percentage of adults (≥15y)	Percentage of household heads that did not complete primary school	Distance to nearest TB clinic (km)	Percentage of HIV prevalence	Percentage of male adults	Year: 2015	Year: 2016	Year: 2017	Year: 2018	Random effects SD: cluster
notification model 1	50.85 (42.97-59.94)	0.96 (0.92-1.00)	0.98 (0.96-0.99)	0.78 (0.69-0.88)	0.99 (0.97-1.02)	0.98 (0.91-1.05)	2.89 (2.48-3.36)	2.91 (2.51-3.38)	2.51 (2.16-2.92)	1.22 (1.03-1.45)	0.32 (0.24-0.40)
notification model 2	49.12 (41.57-57.84)		0.98 (0.97-1.00)	0.82 (0.73-0.91)	0.99 (0.97-1.01)	1.00 (0.94-1.07)	2.89 (2.49-3.37)	2.91 (2.51-3.38)	2.51 (2.16-2.92)	1.22 (1.03-1.45)	0.33 (0.25-0.41)
notification model 3	50.98 (42.89-60.45)	0.98 (0.95-1.02)		0.78 (0.69-0.88)	0.98 (0.96-1.01)	0.98 (0.91-1.05)	2.89 (2.48-3.37)	2.91 (2.51-3.38)	2.51 (2.16-2.92)	1.22 (1.03-1.45)	0.33 (0.26-0.42)
notification model 4	50.11 (42.33-59.15)			0.80 (0.71-0.89)	0.98 (0.96-1.01)	0.99 (0.93-1.06)	2.89 (2.49-3.37)	2.92 (2.52-3.39)	2.51 (2.16-2.93)	1.23 (1.04-1.45)	0.33 (0.26-0.42)
notification model 5	42.17 (36.26-48.91)	1.00 (0.95-1.04)	0.98 (0.96-1.00)		1.00 (0.97-1.02)	0.97 (0.90-1.05)	2.89 (2.48-3.36)	2.91 (2.51-3.38)	2.51 (2.16-2.92)	1.23 (1.03-1.45)	0.37 (0.29-0.46)
notification model 6	42.23 (36.36-49.07)		0.98 (0.96-0.99)		1.00 (0.97-1.02)	0.97 (0.91-1.04)	2.89 (2.49-3.36)	2.91 (2.51-3.38)	2.51 (2.17-2.92)	1.23 (1.04-1.45)	0.37 (0.29-0.46)
notification model 7	42.21 (36.14-49.03)	1.02 (0.98-1.06)			0.99 (0.96-1.01)	0.97 (0.90-1.05)	2.89 (2.49-3.37)	2.91 (2.51-3.38)	2.51 (2.17-2.92)	1.23 (1.04-1.45)	0.39 (0.31-0.48)

Model names	Intercept	Percentage of adults (≥15y)	Percentage of household heads that did not complete primary school	Distance to nearest TB clinic (km)	Percentage of HIV prevalence	Percentage of male adults	Year: 2015	Year: 2016	Year: 2017	Year: 2018	Random effects SD: cluster
notification model 8	42.32 (36.38-49.15)				0.99 (0.96-1.01)	0.95 (0.89-1.02)	2.89 (2.49-3.36)	2.91 (2.51-3.38)	2.51 (2.17-2.92)	1.22 (1.04-1.45)	0.39 (0.31-0.48)
notification model 9	50.76 (42.87-59.71)	0.96 (0.92-1.00)	0.98 (0.96-0.99)	0.78 (0.70-0.88)		0.98 (0.92-1.05)	2.89 (2.49-3.37)	2.91 (2.51-3.38)	2.51 (2.16-2.92)	1.23 (1.04-1.45)	0.31 (0.24-0.40)
notification model 10	48.99 (41.45-57.62)		0.98 (0.97-1.00)	0.82 (0.73-0.92)		1.01 (0.95-1.07)	2.89 (2.49-3.37)	2.91 (2.51-3.39)	2.51 (2.16-2.92)	1.22 (1.03-1.45)	0.32 (0.25-0.41)
notification model 11	50.77 (42.73-60.12)	0.98 (0.95-1.02)		0.78 (0.69-0.89)		0.99 (0.93-1.06)	2.89 (2.49-3.37)	2.91 (2.51-3.38)	2.51 (2.16-2.92)	1.23 (1.03-1.45)	0.34 (0.26-0.42)
notification model 12	49.89 (42.22-58.85)			0.80 (0.72-0.89)		1.00 (0.94-1.07)	2.89 (2.49-3.36)	2.91 (2.50-3.38)	2.51 (2.17-2.92)	1.22 (1.04-1.45)	0.34 (0.26-0.42)
notification model 13	42.19 (36.24-48.95)	1.00 (0.96-1.04)	0.98 (0.96-0.99)			0.97 (0.90-1.04)	2.89 (2.49-3.37)	2.91 (2.51-3.38)	2.51 (2.16-2.92)	1.23 (1.04-1.45)	0.37 (0.29-0.46)
notification model 14	42.21 (36.26-48.83)		0.98 (0.96-0.99)			0.97 (0.92-1.04)	2.89 (2.49-3.37)	2.91 (2.52-3.38)	2.51 (2.17-2.92)	1.23 (1.04-1.45)	0.36 (0.29-0.45)
notification model 15	42.24 (36.28-	1.02 (0.98-1.06)				0.98 (0.91-1.06)	2.89 (2.49-3.37)	2.92 (2.52-3.39)	2.51 (2.16-2.91)	1.23 (1.04-1.45)	0.38 (0.31-0.48)

Model names	Intercept	Percentage of adults (≥15y)	Percentage of household heads that did not complete primary school	Distance to nearest TB clinic (km)	Percentage of HIV prevalence	Percentage of male adults	Year: 2015	Year: 2016	Year: 2017	Year: 2018	Random effects SD: cluster
	49.13)										
notification model 16	42.33 (36.34-49.19)					0.96 (0.90-1.03)	2.89 (2.49-3.36)	2.91 (2.51-3.38)	2.51 (2.17-2.91)	1.22 (1.04-1.45)	0.38 (0.31-0.48)
notification model 17	50.89 (43.09-59.87)	0.97 (0.93-1.00)	0.98 (0.96-0.99)	0.78 (0.69-0.87)	1.00 (0.98-1.02)		2.89 (2.49-3.36)	2.91 (2.51-3.38)	2.51 (2.17-2.91)	1.23 (1.03-1.45)	0.31 (0.24-0.40)
notification model 18	49.14 (41.71-57.90)		0.98 (0.97-1.00)	0.82 (0.73-0.91)	0.99 (0.97-1.01)		2.89 (2.48-3.37)	2.91 (2.51-3.38)	2.51 (2.16-2.92)	1.23 (1.03-1.45)	0.32 (0.25-0.41)
notification model 19	51.11 (42.94-60.52)	0.99 (0.95-1.02)		0.78 (0.68-0.88)	0.99 (0.97-1.01)		2.89 (2.48-3.36)	2.91 (2.51-3.38)	2.51 (2.17-2.92)	1.23 (1.03-1.45)	0.33 (0.26-0.42)
notification model 20	50.35 (42.72-59.25)			0.79 (0.71-0.88)	0.99 (0.97-1.01)		2.89 (2.49-3.37)	2.91 (2.51-3.38)	2.51 (2.17-2.92)	1.22 (1.03-1.45)	0.33 (0.26-0.42)
notification model 21	42.22 (36.22-48.96)	1.00 (0.97-1.04)	0.98 (0.96-1.00)		1.00 (0.98-1.03)		2.89 (2.49-3.37)	2.91 (2.51-3.38)	2.51 (2.17-2.92)	1.23 (1.03-1.45)	0.37 (0.29-0.46)
notification model 22	42.24 (36.26-48.91)		0.98 (0.96-0.99)		1.00 (0.98-1.02)		2.89 (2.49-3.37)	2.91 (2.51-3.38)	2.51 (2.17-2.92)	1.23 (1.04-1.45)	0.37 (0.29-0.46)
notification	42.25	1.03 (0.99-			0.99 (0.97-		2.89 (2.48-	2.91 (2.51-	2.51 (2.16-	1.23 (1.03-	0.39 (0.31-

Model names	Intercept	Percentage of adults (≥15y)	Percentage of household heads that did not complete primary school	Distance to nearest TB clinic (km)	Percentage of HIV prevalence	Percentage of male adults	Year: 2015	Year: 2016	Year: 2017	Year: 2018	Random effects SD: cluster
model 23	(36.19-49.10)	1.06)			1.01)		3.36)	3.39)	2.92)	1.45)	0.48)
notification model 24	42.29 (36.29-49.16)				0.99 (0.97-1.02)		2.89 (2.48-3.36)	2.91 (2.51-3.38)	2.51 (2.16-2.92)	1.22 (1.04-1.45)	0.39 (0.31-0.48)
notification model 25	50.88 (42.99-60.00)	0.96 (0.93-1.00)	0.98 (0.96-0.99)	0.78 (0.69-0.88)			2.89 (2.48-3.37)	2.91 (2.51-3.38)	2.51 (2.16-2.92)	1.23 (1.03-1.45)	0.31 (0.24-0.39)
notification model 26	48.75 (41.40-57.19)		0.98 (0.97-1.00)	0.83 (0.74-0.91)			2.89 (2.49-3.37)	2.91 (2.51-3.39)	2.51 (2.17-2.92)	1.23 (1.03-1.45)	0.32 (0.25-0.40)
notification model 27	50.82 (42.83-60.12)	0.99 (0.95-1.02)		0.78 (0.69-0.88)			2.89 (2.49-3.36)	2.91 (2.52-3.38)	2.51 (2.17-2.92)	1.23 (1.03-1.45)	0.33 (0.26-0.42)
notification model 28	49.89 (42.27-58.58)			0.80 (0.72-0.89)			2.89 (2.48-3.37)	2.91 (2.51-3.38)	2.51 (2.16-2.92)	1.22 (1.03-1.45)	0.33 (0.26-0.42)
notification model 29	42.22 (36.35-48.93)	1.00 (0.97-1.04)	0.98 (0.96-0.99)				2.89 (2.49-3.36)	2.91 (2.51-3.38)	2.51 (2.17-2.92)	1.23 (1.04-1.45)	0.37 (0.29-0.45)
notification model 30	42.22 (36.24-48.94)		0.98 (0.96-0.99)				2.89 (2.49-3.37)	2.91 (2.51-3.38)	2.51 (2.16-2.92)	1.23 (1.03-1.45)	0.36 (0.29-0.45)

Model names	Intercept	Percentage of adults (≥15y)	Percentage of household heads that did not complete primary school	Distance to nearest TB clinic (km)	Percentage of HIV prevalence	Percentage of male adults	Year: 2015	Year: 2016	Year: 2017	Year: 2018	Random effects SD: cluster
notification model 31	42.27 (36.22-49.20)	1.03 (0.99-1.06)					2.89 (2.49-3.37)	2.91 (2.51-3.38)	2.51 (2.16-2.92)	1.23 (1.03-1.45)	0.38 (0.31-0.47)
notification model 32	42.33 (36.38-49.28)						2.89 (2.48-3.36)	2.91 (2.51-3.39)	2.51 (2.16-2.92)	1.22 (1.03-1.45)	0.39 (0.31-0.48)
