# Supplementary material for: Neighbourhood prevalence-to-notification ratios for adult bacteriologically-confirmed tuberculosis reveals hotspots of underdiagnosis in Blantyre, Malawi
Source: PLoS One. 2022 May 23;17(5):e0268749. doi: 10.1371/journal.pone.0268749 (PMC9126376; doi:10.1371/journal.pone.0268749)
Supplement: S3 Table — Coefficients (mean rate ratio) were exponentiated and intercepts were multiplied by 100,000. (PDF) [file pone.0268749.s013.pdf]

S3 Table. Table of all the TB prevalence neighbourhood level models with spatial random effect. Coefficients (mean rate ratio) were exponentiated and intercepts were multiplied by 100,000 (S2 Equation).

Model names	Intercept	Percentage of adults (≥15y)	Percentage of household heads that did not complete primary school	Distance to nearest TB clinic (km)	Percentage of HIV prevalence	Percentage of male adults	Random effects SD: CAR	Probability Zi=0 (Refer to S2 Equation)
prevalence model 33	250.55 (128.71-481.99)	0.91 (0.73-1.13)	0.99 (0.92-1.08)	0.76 (0.42-1.39)	1.02 (0.90-1.14)	1.03 (0.72-1.47)	0.11 (0.01-0.50)	0.18 (0.01-0.45)
prevalence model 34	238.84 (121.98-469.25)		1.01 (0.94-1.08)	0.84 (0.48-1.48)	1.01 (0.90-1.13)	1.07 (0.78-1.49)	0.12 (0.01-0.53)	0.18 (0.01-0.45)
prevalence model 35	252.89 (130.36-480.74)	0.91 (0.75-1.11)		0.78 (0.42-1.42)	1.01 (0.91-1.13)	1.01 (0.71-1.45)	0.13 (0.01-0.53)	0.18 (0.01-0.45)
prevalence model 36	243.80 (126.45-467.87)			0.85 (0.48-1.50)	1.02 (0.91-1.13)	1.08 (0.78-1.49)	0.12 (0.01-0.50)	0.18 (0.01-0.45)
prevalence model 37	214.62 (124.68-375.95)	0.93 (0.76-1.14)	0.99 (0.92-1.07)		1.02 (0.91-1.15)	0.99 (0.70-1.39)	0.11 (0.01-0.47)	0.19 (0.01-0.47)
prevalence model 38	217.74 (128.17-380.36)		1.01 (0.94-1.08)		1.01 (0.90-1.14)	1.03 (0.76-1.39)	0.14 (0.01-0.55)	0.18 (0.01-0.45)
prevalence model 39	218.09 (125.32-385.46)	0.94 (0.78-1.12)			1.02 (0.91-1.14)	0.98 (0.70-1.37)	0.10 (0.00-0.45)	0.18 (0.01-0.46)
prevalence model 40	218.17 (128.15-				1.02 (0.92-1.13)	1.04 (0.79-1.38)	0.11 (0.01-0.45)	0.17 (0.01-0.44)

Model names	Intercept	Percentage of adults (≥15y)	Percentage of household heads that did not complete primary school	Distance to nearest TB clinic (km)	Percentage of HIV prevalence	Percentage of male adults	Random effects SD: CAR	Probability Zi=0 (Refer to S2 Equation)
	370.56)							
prevalence model 41	255.54 (132.62-486.46)	0.91 (0.74-1.12)	1.00 (0.93-1.07)	0.76 (0.41-1.37)		0.99 (0.72-1.37)	0.16 (0.01-0.60)	0.17 (0.01-0.45)
prevalence model 42	244.08 (128.82-462.85)		1.01 (0.95-1.08)	0.85 (0.48-1.48)		1.05 (0.78-1.42)	0.11 (0.00-0.47)	0.17 (0.01-0.44)
prevalence model 43	258.89 (136.73-481.87)	0.91 (0.75-1.10)		0.76 (0.42-1.36)		1.00 (0.73-1.38)	0.11 (0.01-0.45)	0.17 (0.01-0.44)
prevalence model 44	244.95 (128.15-469.47)			0.88 (0.49-1.60)		1.04 (0.76-1.41)	0.11 (0.00-0.47)	0.18 (0.01-0.46)
prevalence model 45	216.07 (126.61-368.62)	0.94 (0.77-1.14)	1.00 (0.93-1.07)			0.96 (0.70-1.30)	0.12 (0.00-0.52)	0.18 (0.01-0.45)
prevalence model 46	218.86 (129.77-367.12)		1.01 (0.94-1.08)			1.00 (0.78-1.30)	0.13 (0.01-0.53)	0.17 (0.01-0.44)
prevalence model 47	221.65 (130.22-380.42)	0.94 (0.78-1.11)				0.95 (0.70-1.28)	0.13 (0.00-0.54)	0.18 (0.01-0.45)
prevalence model 48	221.61 (131.66-378.89)					1.01 (0.78-1.31)	0.11 (0.00-0.47)	0.17 (0.01-0.44)

Model names	Intercept	Percentage of adults (≥15y)	Percentage of household heads that did not complete primary school	Distance to nearest TB clinic (km)	Percentage of HIV prevalence	Percentage of male adults	Random effects SD: CAR	Probability Zi=0 (Refer to S2 Equation)
prevalence model 49	245.92 (127.78-462.08)	0.91 (0.74-1.10)	0.99 (0.92-1.07)	0.78 (0.44-1.39)	1.01 (0.91-1.13)		0.12 (0.01-0.50)	0.17 (0.01-0.44)
prevalence model 50	236.68 (124.73-448.18)		1.01 (0.94-1.09)	0.89 (0.53-1.51)	1.00 (0.90-1.11)		0.11 (0.00-0.47)	0.18 (0.01-0.45)
prevalence model 51	252.77 (131.70-476.30)	0.91 (0.76-1.09)		0.79 (0.43-1.43)	1.01 (0.91-1.12)		0.14 (0.01-0.52)	0.17 (0.01-0.44)
prevalence model 52	234.16 (125.30-435.37)			0.91 (0.55-1.49)	1.01 (0.91-1.11)		0.11 (0.01-0.47)	0.17 (0.01-0.44)
prevalence model 53	215.44 (126.71-366.04)	0.93 (0.78-1.11)	0.99 (0.92-1.07)		1.03 (0.92-1.14)		0.15 (0.01-0.55)	0.17 (0.01-0.45)
prevalence model 54	221.54 (130.86-378.80)		1.01 (0.94-1.08)		1.01 (0.92-1.11)		0.11 (0.00-0.51)	0.17 (0.01-0.44)
prevalence model 55	215.88 (127.63-366.28)	0.94 (0.80-1.10)			1.02 (0.93-1.12)		0.10 (0.00-0.45)	0.17 (0.01-0.44)
prevalence model 56	221.22 (131.40-372.99)				1.01 (0.92-1.11)		0.12 (0.01-0.50)	0.16 (0.01-0.44)
prevalence model 57	257.33 (136.61-	0.91 (0.75-1.09)	1.00 (0.93-1.07)	0.75 (0.42-1.32)			0.10 (0.01-0.43)	0.17 (0.01-0.43)

Model names	Intercept	Percentage of adults (≥15y)	Percentage of household heads that did not complete primary school	Distance to nearest TB clinic (km)	Percentage of HIV prevalence	Percentage of male adults	Random effects SD: CAR	Probability Zi=0 (Refer to S2 Equation)
	472.56)							
prevalence model 58	239.85 (130.33-435.46)		1.01 (0.94-1.08)	0.88 (0.55-1.42)			0.12 (0.01-0.50)	0.17 (0.01-0.44)
prevalence model 59	255.43 (134.76-471.50)	0.91 (0.76-1.08)		0.75 (0.42-1.33)			0.08 (0.01-0.38)	0.16 (0.01-0.43)
prevalence model 60	241.39 (130.69-449.39)			0.90 (0.56-1.44)			0.13 (0.01-0.49)	0.17 (0.01-0.44)
prevalence model 61	217.40 (127.68-373.39)	0.95 (0.81-1.12)	1.00 (0.93-1.07)				0.08 (0.00-0.39)	0.17 (0.01-0.45)
prevalence model 62	226.08 (134.78-389.44)		1.01 (0.94-1.08)				0.14 (0.01-0.51)	0.17 (0.01-0.45)
prevalence model 63	221.43 (131.31-373.68)	0.96 (0.82-1.10)					0.09 (0.00-0.47)	0.17 (0.01-0.44)
prevalence model 64	224.66 (133.79-384.09)						0.09 (0.00-0.44)	0.16 (0.01-0.44)
