# Supplementary material for: Neighbourhood prevalence-to-notification ratios for adult bacteriologically-confirmed tuberculosis reveals hotspots of underdiagnosis in Blantyre, Malawi
Source: PLoS One. 2022 May 23;17(5):e0268749. doi: 10.1371/journal.pone.0268749 (PMC9126376; doi:10.1371/journal.pone.0268749)
Supplement: S4 Table — Coefficients (mean rate ratio) were exponentiated and intercepts were multiplied by 100,000. (PDF) [file pone.0268749.s014.pdf]

S4 Table. Table of all the TB notified neighbourhood level models with spatial random effect. Coefficients (mean rate ratio) were exponentiated and intercepts were multiplied by 100,000 (S1 Equation).

Model names	Intercept	Percentage of adults (≥15y)	Percentage of household heads that did not complete primary school	Distance to nearest TB clinic (km)	Percentage of HIV prevalence	Percentage of male adults	Year: 2015	Year: 2016	Year: 2017	Year: 2018	Random effects SD: CAR
notification model 33	47.05 (39.69-55.57)	0.95 (0.92-1.00)	0.98 (0.96-1.00)	0.87 (0.74-1.02)	1.00 (0.98-1.02)	0.94 (0.87-1.00)	2.89 (2.49-3.36)	2.91 (2.52-3.38)	2.51 (2.16-2.91)	1.22 (1.03-1.45)	0.56 (0.40-0.74)
notification model 34	44.84 (37.88-52.89)		0.99 (0.97-1.00)	0.92 (0.79-1.08)	1.00 (0.98-1.02)	0.96 (0.90-1.03)	2.89 (2.49-3.37)	2.91 (2.52-3.39)	2.51 (2.17-2.92)	1.23 (1.04-1.45)	0.59 (0.43-0.77)
notification model 35	47.90 (40.38-56.69)	0.97 (0.93-1.01)		0.85 (0.72-1.00)	0.99 (0.97-1.01)	0.94 (0.87-1.00)	2.89 (2.48-3.36)	2.91 (2.51-3.38)	2.51 (2.16-2.92)	1.22 (1.03-1.45)	0.59 (0.43-0.77)
notification model 36	46.15 (39.01-54.30)			0.89 (0.77-1.03)	0.99 (0.97-1.01)	0.95 (0.89-1.02)	2.89 (2.49-3.37)	2.91 (2.51-3.38)	2.51 (2.17-2.92)	1.23 (1.04-1.45)	0.60 (0.44-0.78)
notification model 37	42.32 (37.40-47.77)	0.97 (0.93-1.01)	0.98 (0.96-0.99)		1.00 (0.98-1.03)	0.93 (0.86-1.00)	2.89 (2.48-3.35)	2.91 (2.51-3.38)	2.51 (2.16-2.91)	1.22 (1.03-1.45)	0.59 (0.44-0.76)
notification model 38	42.31 (37.31-47.70)		0.98 (0.97-1.00)		1.00 (0.98-1.02)	0.95 (0.89-1.01)	2.89 (2.49-3.37)	2.91 (2.51-3.38)	2.51 (2.17-2.92)	1.22 (1.04-1.45)	0.60 (0.45-0.77)
notification model 39	42.32 (37.35-47.72)	0.99 (0.95-1.03)			0.99 (0.97-1.01)	0.92 (0.86-0.99)	2.89 (2.49-3.37)	2.91 (2.52-3.38)	2.51 (2.16-2.91)	1.23 (1.03-1.45)	0.62 (0.47-0.80)

Model names	Intercept	Percentage of adults (≥15y)	Percentage of household heads that did not complete primary school	Distance to nearest TB clinic (km)	Percentage of HIV prevalence	Percentage of male adults	Year: 2015	Year: 2016	Year: 2017	Year: 2018	Random effects SD: CAR
notification model 40	42.33 (37.31-47.79)				0.99 (0.97-1.01)	0.93 (0.88-1.00)	2.89 (2.49-3.37)	2.91 (2.51-3.39)	2.51 (2.16-2.92)	1.23 (1.04-1.45)	0.62 (0.46-0.79)
notification model 41	47.20 (39.91-55.71)	0.95 (0.92-0.99)	0.98 (0.97-0.99)	0.86 (0.74-1.01)		0.94 (0.88-1.00)	2.89 (2.48-3.36)	2.91 (2.51-3.38)	2.51 (2.16-2.92)	1.22 (1.03-1.45)	0.55 (0.40-0.73)
notification model 42	44.87 (38.02-52.75)		0.99 (0.97-1.00)	0.92 (0.80-1.07)		0.96 (0.90-1.03)	2.89 (2.48-3.36)	2.91 (2.51-3.38)	2.51 (2.17-2.91)	1.22 (1.04-1.45)	0.58 (0.43-0.76)
notification model 43	47.52 (40.04-56.19)	0.97 (0.93-1.01)		0.86 (0.73-1.01)		0.94 (0.88-1.01)	2.89 (2.49-3.35)	2.91 (2.51-3.37)	2.51 (2.16-2.91)	1.22 (1.03-1.45)	0.58 (0.42-0.76)
notification model 44	45.74 (38.85-53.83)			0.90 (0.78-1.04)		0.96 (0.90-1.02)	2.89 (2.49-3.36)	2.91 (2.51-3.38)	2.51 (2.16-2.91)	1.22 (1.04-1.45)	0.59 (0.44-0.77)
notification model 45	42.34 (37.42-47.78)	0.97 (0.93-1.01)	0.98 (0.97-0.99)			0.92 (0.86-0.99)	2.89 (2.49-3.36)	2.91 (2.51-3.38)	2.51 (2.16-2.92)	1.22 (1.03-1.45)	0.58 (0.43-0.75)
notification model 46	42.35 (37.31-47.84)		0.98 (0.97-1.00)			0.95 (0.89-1.01)	2.89 (2.48-3.37)	2.91 (2.51-3.38)	2.51 (2.16-2.92)	1.22 (1.03-1.45)	0.59 (0.44-0.76)
notification model 47	42.35 (37.35-	0.99 (0.95-1.02)				0.93 (0.86-1.00)	2.89 (2.49-3.36)	2.91 (2.51-3.38)	2.51 (2.17-2.92)	1.22 (1.04-1.45)	0.61 (0.45-0.79)

Model names	Intercept	Percentage of adults (≥15y)	Percentage of household heads that did not complete primary school	Distance to nearest TB clinic (km)	Percentage of HIV prevalence	Percentage of male adults	Year: 2015	Year: 2016	Year: 2017	Year: 2018	Random effects SD: CAR
	47.79)										
notification model 48	42.39 (37.43-47.84)					0.94 (0.88-1.00)	2.89 (2.49-3.37)	2.91 (2.52-3.37)	2.51 (2.17-2.92)	1.22 (1.03-1.45)	0.61 (0.46-0.78)
notification model 49	47.94 (40.41-56.77)	0.97 (0.93-1.01)	0.98 (0.96-1.00)	0.85 (0.72-0.99)	1.00 (0.98-1.02)		2.89 (2.49-3.36)	2.91 (2.51-3.38)	2.51 (2.16-2.92)	1.22 (1.03-1.45)	0.57 (0.42-0.75)
notification model 50	45.85 (38.89-53.81)		0.98 (0.97-1.00)	0.90 (0.78-1.04)	1.00 (0.98-1.02)		2.89 (2.48-3.37)	2.91 (2.51-3.38)	2.51 (2.16-2.92)	1.22 (1.04-1.45)	0.59 (0.44-0.77)
notification model 51	48.80 (41.10-57.81)	0.98 (0.94-1.02)		0.83 (0.70-0.97)	0.99 (0.97-1.01)		2.89 (2.49-3.36)	2.91 (2.51-3.38)	2.51 (2.16-2.91)	1.23 (1.04-1.45)	0.61 (0.45-0.79)
notification model 52	47.45 (40.43-55.66)			0.86 (0.75-0.99)	0.99 (0.97-1.01)		2.89 (2.48-3.37)	2.91 (2.51-3.38)	2.51 (2.16-2.92)	1.23 (1.03-1.45)	0.61 (0.45-0.79)
notification model 53	42.27 (37.33-47.71)	0.98 (0.95-1.02)	0.98 (0.96-0.99)		1.01 (0.99-1.03)		2.89 (2.49-3.36)	2.91 (2.51-3.38)	2.51 (2.17-2.92)	1.23 (1.04-1.45)	0.61 (0.46-0.79)
notification model 54	42.29 (37.33-47.75)		0.98 (0.97-1.00)		1.01 (0.99-1.03)		2.89 (2.49-3.37)	2.91 (2.52-3.38)	2.51 (2.17-2.92)	1.22 (1.03-1.45)	0.61 (0.46-0.78)
notification	42.32	1.01 (0.97-			1.00 (0.98-		2.89 (2.48-	2.91 (2.51-	2.51 (2.16-	1.22 (1.03-	0.65 (0.49-

Model names	Intercept	Percentage of adults (≥15y)	Percentage of household heads that did not complete primary school	Distance to nearest TB clinic (km)	Percentage of HIV prevalence	Percentage of male adults	Year: 2015	Year: 2016	Year: 2017	Year: 2018	Random effects SD: CAR
model 55	(37.36-47.74)	1.04)			1.02)		3.36)	3.38)	2.92)	1.45)	0.83)
notification model 56	42.29 (37.29-47.71)				1.00 (0.98-1.02)		2.89 (2.49-3.36)	2.91 (2.52-3.38)	2.51 (2.17-2.92)	1.23 (1.04-1.45)	0.64 (0.49-0.82)
notification model 57	48.29 (40.74-56.87)	0.97 (0.93-1.01)	0.98 (0.97-1.00)	0.84 (0.72-0.98)			2.89 (2.48-3.36)	2.91 (2.51-3.37)	2.51 (2.16-2.92)	1.22 (1.03-1.45)	0.57 (0.42-0.75)
notification model 58	45.99 (39.20-53.76)		0.98 (0.97-1.00)	0.89 (0.78-1.03)			2.89 (2.49-3.37)	2.91 (2.52-3.38)	2.51 (2.16-2.92)	1.23 (1.04-1.45)	0.58 (0.43-0.76)
notification model 59	48.50 (40.78-57.23)	0.98 (0.94-1.02)		0.83 (0.71-0.98)			2.89 (2.49-3.36)	2.91 (2.51-3.38)	2.51 (2.17-2.92)	1.22 (1.04-1.45)	0.60 (0.44-0.78)
notification model 60	47.12 (40.11-55.09)			0.87 (0.76-0.99)			2.89 (2.48-3.37)	2.91 (2.51-3.38)	2.51 (2.16-2.92)	1.23 (1.03-1.45)	0.60 (0.44-0.77)
notification model 61	42.31 (37.32-47.75)	0.99 (0.95-1.03)	0.98 (0.97-1.00)				2.89 (2.49-3.36)	2.91 (2.51-3.39)	2.51 (2.16-2.92)	1.22 (1.03-1.45)	0.61 (0.47-0.79)
notification model 62	42.28 (37.32-47.75)		0.98 (0.97-1.00)				2.89 (2.49-3.37)	2.91 (2.52-3.38)	2.51 (2.17-2.92)	1.23 (1.04-1.45)	0.61 (0.46-0.78)

Model names	Intercept	Percentage of adults (≥15y)	Percentage of household heads that did not complete primary school	Distance to nearest TB clinic (km)	Percentage of HIV prevalence	Percentage of male adults	Year: 2015	Year: 2016	Year: 2017	Year: 2018	Random effects SD: CAR
notification model 63	42.30 (37.29-47.78)	1.01 (0.97-1.04)					2.89 (2.49-3.36)	2.91 (2.51-3.38)	2.51 (2.16-2.92)	1.23 (1.03-1.45)	0.64 (0.49-0.82)
notification model 64	42.33 (37.38-47.84)						2.89 (2.49-3.36)	2.91 (2.51-3.38)	2.51 (2.16-2.92)	1.23 (1.04-1.45)	0.63 (0.48-0.81)
