# Supplementary material for: Neighbourhood prevalence-to-notification ratios for adult bacteriologically-confirmed tuberculosis reveals hotspots of underdiagnosis in Blantyre, Malawi
Source: PLoS One. 2022 May 23;17(5):e0268749. doi: 10.1371/journal.pone.0268749 (PMC9126376; doi:10.1371/journal.pone.0268749)
Supplement: S5 Table — (PDF) [file pone.0268749.s015.pdf]

S5 Table. Table of top ten prevalence models using the ELPD LOO statistic, comparing the models in S1 and S3 Tables.

Model names	ELPD differences	Standard error of differences
prevalence model 32	0.0000000	0.0000000
prevalence model 31	-0.6439814	0.5953633
prevalence model 64	-0.8100483	4.2533603
prevalence model 30	-0.8401287	0.2957389
prevalence model 24	-0.8426507	0.4155533
prevalence model 16	-0.8837806	0.2603595
prevalence model 28	-1.0206936	0.2818817
prevalence model 15	-1.2537159	0.7767991
prevalence model 23	-1.4261711	0.8171422
prevalence model 63	-1.4481648	4.1704611
