# Supplementary material for: Neighbourhood prevalence-to-notification ratios for adult bacteriologically-confirmed tuberculosis reveals hotspots of underdiagnosis in Blantyre, Malawi
Source: PLoS One. 2022 May 23;17(5):e0268749. doi: 10.1371/journal.pone.0268749 (PMC9126376; doi:10.1371/journal.pone.0268749)
Supplement: S6 Table — (PDF) [file pone.0268749.s016.pdf]

S6 Table. Table of top ten notification models using the ELPD LOO statistic, comparing the models in S2 and S4 Tables.

Model names	ELPD difference	Standard error of ELPD difference
notification model 62	0.000000000	0.0000000
notification model 25	-0.006516547	3.2225076
notification model 45	-0.164705190	1.6514375
notification model 61	-0.214042393	0.3547003
notification model 9	-0.228767982	3.3141359
notification model 26	-0.267034261	3.1569474
notification model 17	-0.322702170	3.1960427
notification model 18	-0.340474087	3.0894852
notification model 2	-0.433583819	3.0863202
notification model 46	-0.477146663	1.1087664
