# Supplementary material for: Neighbourhood prevalence-to-notification ratios for adult bacteriologically-confirmed tuberculosis reveals hotspots of underdiagnosis in Blantyre, Malawi
Source: PLoS One. 2022 May 23;17(5):e0268749. doi: 10.1371/journal.pone.0268749 (PMC9126376; doi:10.1371/journal.pone.0268749)
Supplement: S7 Table — Analysis based on post stratified TB prevalence and with confirmed TB notifications kept the same as in the primary analysis. (PDF) [file pone.0268749.s017.pdf]

S7 Table. Parameter estimates for final regression models for predicting neighbourhood level TB prevalence and notifications. Analysis based on post stratified TB prevalence and with confirmed TB notifications kept the same as in the primary analysis.

	Notification model		Prevalence model	
Fixed effects **Parameters**	**Mean rate ratio**	**95% CrI**	**Mean rate ratio**	**95% CrI**
Percentage of adults (≥15y)	0.96	(0.93, 1.00)	1.00	(0.97, 1.02)
Distance to nearest TB clinic (km)	0.78	(0.69, 0.88)		
Percentage of household heads that did not complete primary school	0.98	(0.96, 0.99)		
Year: 2019	Reference			
Year: 2015	2.89	(2.48, 3.37)		
Year: 2016	2.91	(2.51, 3.38)		
Year: 2017	2.51	(2.16, 2.92)		
Year: 2018	1.23	(1.03, 1.45)		
Intercept	$50.88*10^{-5}$	($42.99*10^{-5}$, $60.00*10^{-5}$)	$234.85*10^{-5}$	($219.71*10^{-5}$, $250.68*10^{-5}$)
Zero inflation intercept			0.01	(0.00, 0.05)
Random effects SD: cluster	0.31	(0.24, 0.39)	0.03	(0.00,0.08)

CrI, Credible interval; Km, kilometre; sd, standard deviation.

[a]Percentage of adults was centred by subtracting by its mean (60.90%), Distance to nearest TB clinic (km) was centred by subtracting by 1km, Percentage of household head that did not complete primary school was centred by subtracting by its mean (16.90%).
