# Supplementary material for: Neighbourhood prevalence-to-notification ratios for adult bacteriologically-confirmed tuberculosis reveals hotspots of underdiagnosis in Blantyre, Malawi
Source: PLoS One. 2022 May 23;17(5):e0268749. doi: 10.1371/journal.pone.0268749 (PMC9126376; doi:10.1371/journal.pone.0268749)
Supplement: S8 Table — Analysis based on both microbiologically-confirmed TB and clinically-diagnosed cases and with TB prevalence kept the same as in the primary analysis. (PDF) [file pone.0268749.s018.pdf]

S8 Table. Parameter estimates for final regression models for predicting neighbourhood level TB prevalence and notifications. Analysis based on both microbiologically-confirmed TB and clinically-diagnosed cases and with TB prevalence kept the same as in the primary analysis.

	Notification model		Prevalence model	
Fixed effects **Parameters**	**Mean rate ratio**	**95% CrI**	**Mean rate ratio**	**95% CrI**
Percentage of adults (≥15y)	0.98	(0.95, 1.01)	0.94	(0.80, 1.10)
Distance to nearest TB clinic (km)	0.81	(0.73, 0.90)		
Percentage of household heads that did not complete primary school	0.98	(0.97, 0.99)		
Year: 2019	Reference			
Year: 2015	1.62	(1.47, 1.78)		
Year: 2016	1.82	(1.66, 1.99)		
Year: 2017	1.70	(1.56, 1.86)		
Year: 2018	0.84	(0.76, 0.94)		
Intercept	$163.54*10^{-5}$	($144.68*10^{-5}$, $184.93*10^{-5}$)	$232.08*10^{-5}$	($132.05*10^{-5}$, $404.96*10^{-5}$)
Zero inflation intercept			0.18	(0.01, 0.46)
Random effects SD: cluster	0.30	(0.24, 0.37)	0.33	(0.01, 0.90)

CrI, Credible interval; km, kilometre; sd, standard deviation.

[a]Percentage of adults was centred by subtracting by its mean (60.90%), Distance to nearest TB clinic (km) was centred by subtracting by 1km, Percentage of household head that did not complete primary school was centred by subtracting by its mean (16.90%).
