# Supplementary material for: Neighbourhood prevalence-to-notification ratios for adult bacteriologically-confirmed tuberculosis reveals hotspots of underdiagnosis in Blantyre, Malawi
Source: PLoS One. 2022 May 23;17(5):e0268749. doi: 10.1371/journal.pone.0268749 (PMC9126376; doi:10.1371/journal.pone.0268749)
Supplement: S9 Table — Only neighbourhoods that were included in the 4th quartile were included in the table. (PDF) [file pone.0268749.s019.pdf]

S9 Table. Comparing model results of prevalence to microbiologically confirmed notification rations primary analysis, compared to analysis based on post stratified TB prevalence and based on all TB case notifications. Only neighbourhoods that were included in the 4th quartile were included in the table.

Neighbourhood	TB prevalence to confirmed TB notification ratio	Post-stratified TB prevalence to confirmed TB notification ratio	TB prevalence to all TB notification ratio	TB prevalence to confirmed TB notification ratio quartile	TB prevalence to all TB notification ratio quartile	Post-stratified TB prevalence to confirmed TB notification quartile
1	5.882872	6.961638	1.741540	4	3	4
2	5.835493	6.902129	2.107328	3	4	4
14	7.265753	8.613159	1.874598	4	4	4
15	5.891822	6.865971	1.624834	4	3	4
20	5.640371	6.577177	1.707080	3	3	4
22	6.245970	6.274060	1.841799	4	4	3
23	6.763737	7.160686	1.854707	4	4	4
31	6.696159	7.908075	1.823901	4	4	4
34	6.079875	7.172957	1.682113	4	3	4
37	6.293189	7.377623	1.867094	4	4	4
40	5.391903	5.657743	1.791965	3	4	3
41	5.982987	7.113974	2.239345	4	4	4
44	8.829594	9.244351	2.662622	4	4	4
47	7.544831	8.024778	2.375998	4	4	4
48	6.277072	7.441708	2.262311	4	4	4
52	5.640333	6.596341	1.643353	3	3	4
55	6.184062	6.329324	1.854457	4	4	3
56	5.979641	6.155832	1.857542	4	4	3
57	10.369700	12.219568	2.923005	4	4	4
61	6.253442	7.287852	1.870250	4	4	4
63	6.355325	6.691939	2.239623	4	4	4
64	6.889153	8.139249	2.091773	4	4	4
69	5.218804	6.024331	2.056106	3	4	3
