# Supplementary material for: Neighbourhood prevalence-to-notification ratios for adult bacteriologically-confirmed tuberculosis reveals hotspots of underdiagnosis in Blantyre, Malawi
Source: PLoS One. 2022 May 23;17(5):e0268749. doi: 10.1371/journal.pone.0268749 (PMC9126376; doi:10.1371/journal.pone.0268749)
Supplement: Attachment — Submitted filename: 2022-2-22_Reviewer_Response_McEwenKhundi.docx [file pone.0268749.s020.docx]

**McEwen Khundi**
London School of Hygiene & Tropical Medicine and
Malawi-Liverpool-Wellcome Trust, Blantyre, Malawi

P.O Box 30096

Blantyre

Malawi

Mob: +265 997 615 243
Email: [mcewenkhundi@gmail.com](mailto:peter.macpherson@lstmed.ac.uk)

07/03/2022

Dear Editor, PLOS One

Thank you for the helpful review of our manuscript. Please find below our detailed response to all the queries that we were required to address.

Yours Sincerely,

Mcewen Khundi

(On behalf of the Authors)

Editor Comments to Author:

Journal Requirements:

1. When submitting your revision, we need you to address these additional requirements.

Please ensure that your manuscript meets PLOS ONE's style requirements, including those for file naming. The PLOS ONE style templates can be found at 
<https://journals.plos.org/plosone/s/file?id=wjVg/PLOSOne_formatting_sample_main_body.pdf> and 
<https://journals.plos.org/plosone/s/file?id=ba62/PLOSOne_formatting_sample_title_authors_affiliations.pdf>

Thank you, we have now followed these formatting guidelines throughout.

2. Thank you for stating the following in the Acknowledgments Section of your manuscript:

(This work was supported by two grants from Wellcome Trust (ELC grant number WT200901/Z/16/Z) and (PM grant number 200901/Z/16/Z). JRC was funded by UK Medical Research Council (MRC) programme grant MC_UU_00004/07. PJD was supported by a fellowship from the MRC (MR/P022081/1); this UK funded award was part of the EDCTP2 programme supported by the European Union. RMB was funded by Wellcome Trust (203905/16/Z). KCH was supported by the European Research Council (757699) and UK FCDO (Leaving no-one behind: transforming gendered pathways to health for TB). The funders had no role in study design, data collection and analysis, decision to publish, or preparation of the manuscript.)

We note that you have provided funding information that is not currently declared in your Funding Statement. However, funding information should not appear in the Acknowledgments section or other areas of your manuscript. We will only publish funding information present in the Funding Statement section of the online submission form. Please remove any funding-related text from the manuscript and let us know how you would like to update your Funding Statement. Currently, your Funding Statement reads as follows:

(This work was supported by two grants from Wellcome Trust <https://wellcome.org/> (ELC grant number WT200901/Z/16/Z) and (PM grant number 200901/Z/16/Z). JRC was funded by UK Medical Research Council  (MRC) programme grant <https://www.ukri.org/councils/mrc/guidance-for-applicants/types-of-funding-we-offer/programme-grant/> MC_UU_00004/07. PJD was supported by a fellowship from the MRC (MR/P022081/1); this UK funded award was part of the EDCTP2 programme supported by the European Union. RMB was funded by Wellcome Trust (203905/16/Z). KCH was supported by the European Research Council  <https://erc.europa.eu/> (757699) and UK FCDO (Leaving no-one behind: transforming gendered pathways to health for TB)  <https://www.gov.uk/government/organisations/foreign-commonwealth-development-office/about/research>. TC was supported by US NIH R01 R01AI147854.
The funders had no role in study design, data collection and analysis, decision to publish, or preparation of the manuscript.)
Please include your amended statements within your cover letter; we will change the online submission form on your behalf.

>> Thank you. We have included the funding information in the cover letter and have removed it from the manuscript.

3. We note that you have stated that you will provide repository information for your data at acceptance. Should your manuscript be accepted for publication, we will hold it until you provide the relevant accession numbers or DOIs necessary to access your data. If you wish to make changes to your Data Availability statement, please describe these changes in your cover letter and we will update your Data Availability statement to reflect the information you provide.

>> We have now updated the repository access settings, meaning it is now publicly accessible.

https://github.com/mcewenkhundi/TBhotspotclustersplos

4. We note that Figures 1, 2, and 4 in your submission contain map images which may be copyrighted. All PLOS content is published under the Creative Commons Attribution License (CC BY 4.0), which means that the manuscript, images, and Supporting Information files will be freely available online, and any third party is permitted to access, download, copy, distribute, and use these materials in any way, even commercially, with proper attribution. For these reasons, we cannot publish previously copyrighted maps or satellite images created using proprietary data, such as Google software (Google Maps, Street View, and Earth). For more information, see our copyright guidelines: <http://journals.plos.org/plosone/s/licenses-and-copyright>.
 
We require you to either (1) present written permission from the copyright holder to publish these figures specifically under the CC BY 4.0 license, or (2) remove the figures from your submission:

a. You may seek permission from the original copyright holder of Figures 1,2, and 4 to publish the content specifically under the CC BY 4.0 license.  
 
We recommend that you contact the original copyright holder with the Content Permission Form (<http://journals.plos.org/plosone/s/file?id=7c09/content-permission-form.pdf>) and the following text:
“I request permission for the open-access journal PLOS ONE to publish XXX under the Creative Commons Attribution License (CCAL) CC BY 4.0 (<http://creativecommons.org/licenses/by/4.0/>). Please be aware that this license allows unrestricted use and distribution, even commercially, by third parties. Please reply and provide explicit written permission to publish XXX under a CC BY license and complete the attached form.”
 
Please upload the completed Content Permission Form or other proof of granted permissions as an "Other" file with your submission.
 
In the figure caption of the copyrighted figure, please include the following text: “Reprinted from [ref] under a CC BY license, with permission from [name of publisher], original copyright [original copyright year].”
 
b. If you are unable to obtain permission from the original copyright holder to publish these figures under the CC BY 4.0 license or if the copyright holder’s requirements are incompatible with the CC BY 4.0 license, please either i) remove the figure or ii) supply a replacement figure that complies with the CC BY 4.0 license. Please check copyright information on all replacement figures and update the figure caption with source information. If applicable, please specify in the figure caption text when a figure is similar but not identical to the original image and is therefore for illustrative purposes only.
The following resources for replacing copyrighted map figures may be helpful:
 
USGS National Map Viewer (public domain): <http://viewer.nationalmap.gov/viewer/>
The Gateway to Astronaut Photography of Earth (public domain): <http://eol.jsc.nasa.gov/sseop/clickmap/>
Maps at the CIA (public domain): <https://www.cia.gov/library/publications/the-world-factbook/index.html> and <https://www.cia.gov/library/publications/cia-maps-publications/index.html>
NASA Earth Observatory (public domain): <http://earthobservatory.nasa.gov/>
Landsat: <http://landsat.visibleearth.nasa.gov/>
USGS EROS (Earth Resources Observatory and Science (EROS) Center) (public domain): [http://eros.usgs.gov/#](http://eros.usgs.gov/)
Natural Earth (public domain): <http://www.naturalearthdata.com/>

>> In Figure 1 and Figure 2, we used study-defined map boundaries, captured by our Study Research Assistants. As we generated these maps, we are happy for them to be distributed under a CC-BY license. No other copyright-protected material is included.

>> In Figure 4, we used an OpenStreetMap background map tile, made freely available for reuse under a permissive CC-4.0 license. We have checked with the OpenStreetMap Foundation, and they have confirmed that no permission is required to reuse or republish these map tiles, although they cannot provide written confirmation of this (<https://www.openstreetmap.org/copyright>). We have added text to the footnote of Figure 4 to ensure attribution is correctly worded in accordance with the OpenStreetMap Foundation guidelines, below:

- https://wiki.osmfoundation.org/wiki/Licence/Community_Guidelines
- https://wiki.osmfoundation.org/wiki/Licence/Licence_and_Legal_FAQ#What_about_map_tiles?
- <https://wiki.osmfoundation.org/wiki/Licence/Community_Guidelines/Produced_Work_-_Guideline>
- https://wiki.osmfoundation.org/wiki/Licence/Attribution_Guidelines

5. Please include captions for your Supporting Information files at the end of your manuscript, and update any in-text citations to match accordingly. Please see our Supporting Information guidelines for more information: <http://journals.plos.org/plosone/s/supporting-information>.

We have provided captions as requested.

**Reviewer(s)' Comments to Author:**
**Referee: #1**

 In this manuscript, Khundi and colleagues explore the relationship, at a neighborhood level, between TB case notification ratio, TB prevalence, and a short list of demographic and health care access variables. They highlight the important distinction between an area’s TB notification rate and its burden of undiagnosed prevalent TB, and they demonstrate that the ratio of these two quantities varies in ways that might be expected based on access to health care. The work is overall important, rigorously performed, and in my opinion a valuable addition to PLOS One. But I also think that the work would be considerably strengthened by clarifying several aspects of the methods, results, and interpretation, as follows.

Methods:

1. Aspects of methods that need to be more clearly described:
2. In the prevalence survey, what testing was done on “confirmatory specimens”, and how was a prevalent case defined?

>> Thank you for bringing this up. We have added the definition that was used for a prevalent case in the study. We have deleted the part that mentioned “confirmatory specimen” because this procedure was already described under the section “**Blantyre enhanced TB monitoring and evaluation of TB notifications**”

We have added this text to the manuscript to describe the case definition line 139 to line 141:

“a prevalent TB case was defined as a positive result for Xpert MTB/Rif or smear microscopy, with a positive MGIT culture result that was speciated as *Mycobacterium* *tuberculosis*.”

3. Please describe how NSO 2008 and 2018 census data were used to extrapolate the neighbourhood populations from 2015 to 2019. Was a single growth rate assumed for all age groups, for example? Also clearly state the assumptions made, e.g. that growth was (presumably) assumed to be the same in all neighborhoods.

>> The extrapolation of the neighbourhood denominators was based on the study census of 2015 and the 2018 NSO census. We have removed the reference to 2008 census, which was incorrect. The growth rates were calculated separately for ages 0 to 4yrs, 5 to 14 yrs, 15yrs or older adult males and 15yrs or older adult females. The growth rate was assumed to be the same for all the neighbourhoods.

Below is the edited text from manuscript reflecting the changes made, refer to line 148 to line 156:

“In 2015, the MLW study team conducted a population census in all of the city’s CHW areas [1]; in an independent exercise the Malawi National Statistical Office (NSO) conducted the Malawi Population and Household National Census in 2018 that included Blantyre City [2]. Population denominators were derived from the MLW study team's 2015 census and the NSO’s 2018 census. The 2018 NSO and 2015 MLW study census data were used to calculate annual population growth rates, which were then used to estimate annual population denominators for the 72 study neighbourhoods from 2015 to 2019. The growth rates were calculated separately for ages 0 to 4yrs, 5 to 14yrs, 15yrs or older adult males and 15yrs and older adult females. Growth rates were assumed to be the same for all the neighbourhoods.”

4. How are the various years’ notification data accounted for in the CNR model? Table 2 suggests that all years’ data are included, but it’s not clear to me which variables in Equation 1 correspond to different years’ notifications.

>> There was a fixed effects term for the year. Below I have included the model from the text to illustrate the model structure.

We have modified the equation to clearly show how the years were included in the model line 207 to line 224.

“**Equation 1**

Let $Y\sim Pois\left( \mu\right)$

$$Pr(Y_{ij}=y_{ij})=\left( \frac{\mu_{ij}^{y_{ij}} exp(-\mu_{ij})}{y_{ij}!} \right)$$

$$\log\left( \mu_{ij} \right)= \alpha+\alpha_{i}+ \beta_{1}x_{1i}+ \beta_{2}x_{2i}\ldots+\beta_{k}x_{ki} + \beta_{year2015}{year2015}_{ij}+ \beta_{year2016}{year2016}_{j}+ \beta_{year2017}{year2017}_{j}+\beta_{year2018}{year2018}_{j}+ \log\left( {Pop}_{ij} \right)$$

$$\alpha\sim Normal\left( \mu_{\alpha}=0,\sigma_{\alpha}^{2}=10 \right)$$

$$\beta_{k} \sim Normal\left( \mu_{\beta}=0,\sigma_{\beta}^{2}=10 \right)$$

$$\alpha_{i} \sim Normal\left( 0,\sigma_{\alpha_{i}}^{2} \right)$$

$$\sigma_{\alpha_{i}} \sim HalfCauchy\left( 0, 1 \right)$$

The expectation of $Y_{ij}=y_{ij}$ given by:

$$E(Y_{ij}=y_{ij})= \mu_{ij}$$

Where i refers to neighbourhood i for i =1,2,3..72, and *j* indexes year (2015,…2019), ($\alpha+\alpha_{i}$) intercept parameters and $\beta_{1}, \beta_{2}\ldots\beta_{k}$ are unknown regression coefficients that are estimated from the data for the cluster level covariates $x_{1i}, x_{2i}\ldots$ $x_{ki}$, and sigma ($\sigma_{\alpha_{i}}$) is the standard deviation for the random intercept for neighbourhood i. ${Pop}_{ij}$ is the total population of neighbourhood in year *j* that is used as the offset. Here ${year2015}_{j}$, ${year2016}_{j}$, ${year2017}_{j}$ and${year2018}_{j}$ are dummy variables which take the value of one for that year and take the value of zero for the other years (the baseline year was 2019).

”

5. Is the PNR based only on the estimated CNR (and prevalence) in 2019? I assume this is the case, but I don’t see it explicitly stated in the Methods.

>> No, we used CNRs data from 2015-2019 for each of the 72 areas to estimate the model. We included a dummy variable for year, with the reference level of 2019. In this way all the data contributed to estimating the coefficients and in obtaining the predicted PNR for each of the 72 areas in 2019.

We have edited the manuscript to clarify this point as follows (see line 189 to line 192):

“The bacteriologically-confirmed case notification data were modelled using a Poisson response distribution (Equation 1), we included a dummy variable for year with the reference level of 2019.”

Also refer to the modified Equation 1 on the query above.

6. The specification of the zero-inflated prevalence model (Equation 2) is unclear to me. First, what is meant by “(so W = 0 )” and “(so Z > 0 )” in line 233? ~ (1 − ) generates some zero values for Z, but it’s unclear why this implies anything about the value of W; and similarly, ~ () will require W (but not Z) to be >0. I wasn’t sure if Z and W were reversed here, or “so” was the wrong word, or there’s something else about the model specification that I’m not understanding. Also in equation 2, In line 237, there seem to be too many “=”s. After the first curly bracket, what is it that is “= 0” or “> 0”? Is there a variable or condition missing?

>>Thank you for spotting the mistake and apologies for the confusion.

We have corrected Equation 2 (line 225 to line 246) as shown below and hopefully this is now clear.

“**Equation 2**

To define the zero-inflated Poisson model, let $Z\sim Bern(1-p)$ (so Pr{Z = 0}=*p*) and independently $W\sim Pois\left( \mu\right)$. Then the data are modelled by the zero-inflated variable Y which is defined as$Y=ZW$. Therefore have:

$$Pr(Y_{i}=y_{i})=\left\{ \begin{aligned} \left( p+\left( 1- p \right)exp\left( -\mu_{i} \right) \right) if y_{i}=0 \\ \left( \left( 1-p \right)\frac{\mu_{i}^{y_{i}} exp(-\mu_{i})}{y_{i}!} \right) if y_{i}\geq1. \\ \end{aligned} \right.$$

(where *i=1,…72,* indexes cluster (note we only have prevalence data for one year, 2019).”

7. I had a couple of questions about the chosen covariates:
8. Is primary school completion known to be a reliable indicator of poverty in this setting? If not, then it would be useful to see consideration of alternative proxies.

>> We chose the percentage of household heads who did not complete primary school as a proxy of poverty because a lack of basic education in the household head is strongly associated with poverty [3]. As the figure below shows, the Malawi Integrated Household Survey found that household head completion of primary education is a good predicator of household poverty [4].

Figure copied from Malawi Integrated Household Survey 2010-2011 report page 222 [4].

The following text has been added to the manuscript line 169 to line 173:

“… the percentage of households whose head never finished primary school — a proxy variable for poverty — was estimated using data from the TB prevalence study. According to the Malawi Integrated Household Survey household head education level was closely associated with household poverty [24,25]”

9. Why would the percentage of the population who were adults be expected to predict PNR? Or is this likely to be a proxy for some other characteristic of importance, such as geographic mobility or proximity to city center?

>>. Age is a strong predicator of TB prevalence and notifications. Children <15 years are less likely to have prevalent or notified TB. Our logic was that areas with higher percentages of adults would have more prevalent TB or notified TB.

10. In general, why was this particular set of covariates chosen?

>> The set of variables considered for inclusion in the model were selected based on careful consideration of their likely predictive ability, their ease of measurement within programmatic settings such as Malawi [1], and their availability in both the prevalence and notification datasets. Given these variables, and drawing on our previous work [1], we undertook statistical model selection to select the most parsimonious predictive model (S1-S6 Tables).

We have edited the text in line 196 to line 202 to provide greater description:

“Possible combination of neighbourhood-level variables were derived for the TB case notification and prevalence data. The set of variables considered for inclusion in the model were selected based on previous research [1] ,ease of measurement for TB programmes, and availability within the datasets. The models' predictive performance were evaluated using leave-one-out (LOO) cross-validation [5]. The best fitting models were selected based on their expected log pointwise predictive density (ELPD) LOO statistic (S5 and S6 Tables) [5].

11. Minor methods comments:
- Abstract Methods and findings: Make clear that the second sentence describes the linked prevalence survey.

>> Thank you, we have edited the sentence in line 29 to line 32

“In the prevalence survey, consenting adults from randomly selected households in 72 neighbourhoods had symptom-plus-chest X-ray screening, confirmed with sputum smear microscopy, Xpert MTB/Rif and culture.”

12. Github link doesn’t yet work.

>> Apologies. The GitHub link was previously private whilst we conducted analysis, but now it has been changed to be publicly accessible.

https://github.com/mcewenkhundi/TBhotspotclustersplos

Results:
A key finding is that PNR varied by neighbourhood, and in ways that were only partially explained by the measured covariates. But to really understand these results and their implications, there are several relationships that I would like to see described or quantified:
13. Can you more clearly characterize to what extent CNR was predictive of prevalence? Statistically, this might require a single model that examines the association between CNR and prevalence. It would also be useful to see a visual representation of the CNRs and the corresponding PNRs. Figure 2 may be an attempt to show this, but it’s difficult to distinguish the color scales in the small geographic areas. One possibility is to use color in figure 3 not to show quartile (which can already be determined from location on the figure) but to show either the absolute prevalence or the absolute CNR.

>> Our analysis seeks to do this, but by focusing on the Prevalence-Notification Ratio (PNR), because this measure highlights areas of under-reporting, which are important to identify for planning where (and how) to intervene. We chose to use separate models for prevalence and notification because we had two separate data sets. It also enabled us to have a greater control over understanding predictors influence, and adequate model building work by keeping each separately.

Thank you for the suggestion for modifying this figure – we have done as you have suggested and we agree that this change improves the clarity of the message.

14. Similarly, of the variation in PNR, can you quantify how much was explained by the measured covariates and how much was modelled as neighborhood level random effects?

>> As we modelled notifications and prevalence datasets separately, we consider this for each model in turn.

From Table 2 the random effects standard deviation for the Case Notification Rate model was 0.31 (0.24, 0.39), after adjusting for significant covariates. This is quite a tight interval, and well away from zero, consistent with highly significant neighbourhood level variation after allowing for covariates. For the prevalence model the estimated random effects standard deviation was 0.33 (0.01, 0.90). This interval is wider, as there is less information in the prevalence data, but suggests a similar level of neighbourhood variation after adjusting for covariates.

15. The trend in annual CNR is notable. Was there any relationship between the rate of decline in CNR (which might suggest greater access to care, or a temporary increase in notifications out of proportion to the prevalence ) and the 2019 PNR?

>> Thanks for this good suggestion, which we have interrogated further. We found that case notification rates declined linearly over time. This is not explained by the covariates in the CNR model because the trend is still present even after adding the covariates. Hence, it is unlikely to be explained by access to care. And also while this is interesting we feel like pursuing it might end up stretching the word limit too much.

16. Lines 296-297, “The rates of empirical TB notifications were higher in the city centre and were lower in the city’s outskirts, whereas empirical TB prevalence rates

were higher on the outskirts of the city”: Is there any way to characterize this relationship numerically, i.e., how strong and how consistent were these patterns?

>> This observation is correct and important.

In our models, we investigated the association between distance from neighbourhoods and the nearest clinic. This was because, both for TB notifications and prevalence, access to clinical services is an important determinant of underdiagnoses. We found that clinic distance was an important predictor of notification rates. The reviewer is correct that P:N ratios were higher in the peripheral areas of the city, which we know to be comprised of informal settlements. However, we think this pattern is probably determined by a broader set of determinants, including access to clinics, poverty, and potentially higher transmission. Unfortunately, it is very difficult to capture this in a single metric. Therefore, we believe that our model which incorporates clinic distance, and a marker of poverty (household head education level) best captures this component.

We realise that this is important and in our current research, we are actively exploring ways on how we can measure “informality”.

We have added text to the discussion section emphasise this point in line 380 to line 383:

“and further research is required to quantitatively define “the degree of formality of settlement”; these are residential areas that are not registered by the authorities or have makeshift housing structures, and are likely to be important areas of focus for TB case finding activities.”

17. Due to small numbers of prevalent cases, uncertainty in the prevalence is wide, and it’s not clear how this affects the main results. How confident can we be that the neighborhood level prevalence estimates improve the identification of high-prevalence neighborhoods, compared to predictions based on neighborhood demographics? Or alternatively, how densely would prevalence surveys need to sample individual neighborhoods, in order to have reasonable certainty of improving on demographic-based models?

>> Thanks – this is a very good point. By way of background, prevalence surveys are extremely costly and resource intensive, and very few have been conducted at the city scale. Based on what we found in our prevalence survey, predictive models are unlikely to be anywhere near good enough because we only found one good predictive covariate, even with a relatively large sample size (compared to subnational data available in National TB Prevalence surveys). In addition, to the best of our knowledge, there is no established model based on covariates that predicts prevalence. Indeed, in a recent publication from our group using subnational prevalence survey data from Pakistan, five separate research groups were unable to identify a unifying set of predictors for prevalence [6]).

We have edited and added text to the Discussion to emphasise these points. Line 452 to line 455

“The prevalence survey detected a low number of prevalent cases in the city. This meant that the models for prevalence rate had less power for identifying predictive covariates. Other work modelling regional TB prevalence has also found most potential covariates were unable to improve predictions [6].”

18. In practice, if notification data plus neighborhood characteristics will be used to guide case finding, then only prior years’ notification data will be available at the time of screening site selection. If 2019 CNRs were not yet available, how would the selected model have predicted 2019 prevalence from 2015-2018 notification data?

>>

We have a model of PNR that uses data that we could collect in a single year and allows us to make a prediction without reliance on the prior year’s CNR. The value of the prior years in estimating the CNR model is to increase the efficiency of the CNR model estimates through including the additional data. Once we estimate the CNR model we do not need the data from the previous year in order to estimate the CNR for a new region.

19. It looks like the mean distance from neighborhood centroid to the nearest clinic (1.7 km) is comparable to the size of many of the neighborhoods shown in Figs 1-2 -- suggesting that the average distance may not be a good estimate of the distance to clinic for much of a neighborhood. If this is the case, I recommend (1) evaluating how well distance to clinic from a neighborhood’s center predicts the distance to clinic for each of that neighborhood’s residents, and (2) considering whether population density or distance to city center may be a more appropriate neighborhood-level covariate than distance to health facility.

>> Thanks to the Reviewer for this point. In response, we note that the mean is not the best statistic for measuring distance from the neighbourhood centroid to the nearest clinic given the non-symmetric distribution of the distances (see histogram below). Further, our previous work showed that population density was not associated with notifications in this setting [7].

More minor, some aspects of the results that should be more clearly stated:
20. Fig 2: High max values of color scales makes variation difficult to see.

>> Fig 2 has been replaced, thank you.

21. 60.9% adults estimate: is this in 2015 or 2019? (Or if this was measured in 2015 and assumed to also apply in 2019, this should be stated more clearly in the methods as noted above.)

>> This was from the study census in 2015 and it was assumed to apply in 2019 based on our population projections.

Below is text in the manuscript that mentions the source of each variable with minor edits to clarify the point above to clarify this line 163 to line 173.

“The percentage of adults in each neighbourhood and the percentage of male adults aged 15 years or older were calculated using data from the 2015 study team Blantyre City census and were assumed to be consistent from 2016 to 2019. The distance to the nearest TB clinic was estimated by calculating the cartesian (straight line) distance between the centroid of each neighbourhood and the nearest TB clinic; this served as a proxy indicator for access to TB diagnosis and treatment. Neighbourhood HIV prevalence was calculated using prevalence survey data, and the percentage of households whose head never finished primary school — a proxy variable for poverty — was estimated using data from the TB prevalence study. According to the Malawi Integrated Household Survey, household head education level was closely associated with household poverty [3,4]. “

The percentage of adults was estimated from the 2015 population but was assumed to be consistent within the projected/extrapolated 2019 population. Here we compare the 2015 estimate 60.90 % (range: 54.80-70.60, sd: 3.00) versus the newly calculated based on the projected 2019 population 62.8 % (range: 56.9-72.3, sd: 2.93).

22. What is meant by the “midpoint of the centre of the cluster”?

>> Thank you. We have changed the phrase “midpoint of the centre of the cluster” to “centroid of the neighbourhood”; the centroid is the geometric mean centre point of a spatial feature.

We have changed this in the manuscript lines 288 to lines 289:

“The mean distance from the centroid of the neighbourhoods to the nearest TB clinic was (1.73 km, range: 0.36-3.66 km, sd: 0.88) Table 1”

23. Table 1: Why “Estimated mean HIV prevalence” and not just “HIV prevalence” or “estimated HIV prevalence”?

>> We have reworded “Estimated mean HIV prevalence” in Table 1 to “HIV prevalence” and we have also changed at other points where this phrase was used. This was an empirical measure of the mean neighbourhood HIV-prevalence using prevalence survey data.

24. Figure 1 (and similar): Please provide a legend for reference showing distance in km, as distances to clinic are given in km and it will be difficult for most readers to convert degrees longitude/latitude to distance.

>> Thank you for this suggestion. We have done this.

Figure1 with distance scale

Figure2 with distance scale

25. Consider including units (“years”) for PNR, to convey more clearly that this is a ratio divided by an annual rate. Related to this, I would not call PNR a “rate ratio.” (Although “ratio” isn’t entirely accurate either, at least it’s more standard).

>> We will refer to it as prevalence to notification ratio and drop the “rate” word. We do acknowledge that PNR has the unit of years but for this analysis we have opted not to use it.

See our acknowledgement of PNR units of “years” in the discussion line 411 to line 413:

“The P:N ratio, which is used to assess TB burden in this study, is a proxy for the time between onset of TB infectiousness and diagnosis, and it is typically given the unit of years in mathematical modelling studies; its inverse is known as the patient diagnostic rate [8,9].”

In the manuscript we have changed all places that mentioned prevalence to notification rate ratio to “prevalence to notification ratio”, abbreviated to P:N ratio.

Objectives, discussion, and interpretation:

26. Within the abstract, the results presented don’t support the conclusion that neighborhood prevalence surveys provide more information about hotspots than could be predicted by neighborhood socioeconomic characteristics or health center locations. Both in the abstract and throughout the paper, this is a key question that should be addressed head-on but isn’t.

>> The reviewer is correct that this is a central observation from our analysis, and from our prior beliefs, we would have expected that prevalence data provides additional data about underdiagnosis which is not available from notifications and neighbourhood characteristics alone. Our main objective was to investigate whether prevalence and notification data could be combined to reliably identify areas of underdiagnosis. In addition without prevalence data it would not be possible to know whether a low CNR meant there wasn’t much TB, or there was poor access to care. The prevalence data lets us distinguish these possibilities. Without it, one would hope that one’s preconceptions about predictor variables were correct; with it, we have been able to establish the actual relationship.

27. Relatedly, throughout the manuscript, it is unclear whether the authors are offering their finding as a basis for identifying high-prevalence neighborhoods in other settings, or whether they are suggesting that every city needs to do its own neighborhood-level prevalence survey and similar modeling analysis. This is ambiguous in the introduction lines 77-81 (which actually suggests a third option that I don’t think was intended – that they developed a model which others can then use to identifying neighborhood determinants of underdiagnosed TB in Blantyre.) There’s also a seeming contradiction starting in line 400, where the authors acknowledge resource constraints that limit prevalence surveys, yet they suggest that prevalence surveys should be conducted to improve the targeting of case-finding interventions. Please clarify the intended objective, and discuss (1) whether findings demonstrate with any certainty that a prevalence survey adds information, beyond what could be predicted using notifications plus “poverty and population density”, and (2) whether any added value of the prevalence survey is sufficient to warrant the expense.

>> Thank you for raising this, we agree this was not clear. Yes, it is our intention that that our approach provides a tool which we can use in other similar urban settings to relatively cheaply identify areas likely to have the greatest under-diagnosis of TB (yes you are of course correct that use of this model in Blantyre is not strictly needed as we have both the notification and the prevalence survey data — nevertheless the model still usefully smooths the data). Hence the need of carrying out a prevalence survey might be reduced but may still be appropriate for other purposes – not least validating the model predictions, and further developing and improving our approach.

In the introduction section I have edited the text below line 80 to line 84:

“TB prevalence surveys are, however, costly and logistically demanding. Identification of neighbourhoods with high levels of under-diagnosis without requirement for prevalence surveys could be of major benefit to National TB Programmes. Here, we aimed to develop simple, accurate models that could be used by researchers and TB Programme Managers using high-quality spatially-resolved TB neighbourhood level data from urban Blantyre, Malawi.”

We have added the following text to the discussion section line 398 to line 39402:

“But, by using the covariates identified in our models, our hope is that new setting with similar characteristics to Urban Blantyre might successfully rank its neighbourhoods to identify areas likely to have underdiagnosis. Hence our approach may reduce the need to carrying out a relatively expensive across-the-board prevalence survey [6].

”

28. Please discuss the unusually rapid changes in Blantyre’s CNR between 2015 and how this might affect the results. For example, recent intensification of case finding efforts might temporarily increase notifications out of proportion to the impact on prevalence, resulting in a lower PNR. Can you comment on what was driving these notification trends in Blantyre and how it might have differed by neighborhood?

>> Thank you for this comment. Many other African settings have experienced a similar trends of reductions in case notification rates [10]. In addition Malawi (especially Blantyre) has done very well because of very high coverage of HIV treatment and roll out of isoniazid preventive therapy (IPT) with high coverage [11]. HIV treatment was also universally high across neighbourhoods. We agree that neighbourhood-level trends are interesting, and will be investigated in separate analyses.

We have added the text below in the manuscript discussion section line 408 to line 410:

“CNRs in Blantyre Malawi declined between 2015 to 2019, reflecting the general trend in other African countries in this period [10]. Blantyre has also achieved high coverage of HIV treatment and isoniazid preventive therapy [9].”

29. Please discuss the relevance of the selected covariates versus others that might have been considered in such a model.

>> Thanks – addressed in response to Referee #1 query 10.

**Referee: #2**

1. An improved ability to identify detailed geographical areas where underdiagnosis of TB is occurring is critical for efficient resource allocation in Malawi and could be applied in other urban settings. This work provides a useful tool to refine estimates of undiagnosed TB prevalence at a local level.

>> Thank you so much for your positive feedback

2. I suggest the sparseness of the prevalence data could be highlighted more as part of the context to highlight the need for this approach, as detailed prevalence data would make underdiagnosis easily determined. It was not apparent until reviewing Table 1 and being able to see that just 29 adults across the 72 neighbourhoods had bacteriologically confirmed TB cases during the 2019 survey, leaving an extremely wide uncertainty range for true undiagnosed TB prevalence, relative to the hundreds of case notifications annually through regular screening.
   >> Yes this is a limitation. But prevalence surveys are extremely resource intensive and expensive. These are the data that we had available for modelling; future studies may be able to do more with a larger prevalence survey sample size (possibly by combining with our data). Please also see our response to a similar question by Reviewer#1 query 17.

We have acknowledged these limitations in several places:

The prevalent cases found were described in the results section line 308 to line 309 :

“A total of 29 (range: 0-3, sd: 0.6) bacteriologically-confirmed previously undiagnosed adult TB patients were identified during the prevalence survey”

Between line 452 and line 454 in the discussion section:

“The prevalence survey detected a low number of prevalent cases in the city. This meant that the models for prevalence rate had less power for identifying predictive covariates.”

3. It would be extremely valuable to give some context for why case notification rates have dropped from 2015 to 2019 in these neighbourhoods – based on WHO estimates and other modelling I’m familiar with this may primarily be because of drop in TB incidence (and prevalence) over this period due to scaled up TB interventions rather than a reduction in notification rates. This is especially important as the year has such a high fixed rate as part of the TB notification model in Table 2 so it seems at least naively like changes in notifications by year could be captured on the prevalence side rather than the notification side, which might change the conclusions substantially.
   >> Thank you. Addressed in response to Reviewer 1 query number 28.

We have added the text below in the manuscript discussion section line 408 to line 409:

“CNRs in Blantyre Malawi declined between 2015 to 2019, reflecting the general trend in other African countries in this period [10]. Blantyre has also achieved high coverage of HIV treatment and isoniazid preventive therapy [9].”

4. It could be discussed that a high P:N ratio alone is not necessarily a case for prioritized spending as the characteristics that define a “high underdiagnosis” neighbourhood are largely independent of TB prevalence, and rather that the estimate of undiagnosed prevalence given the estimated P:N ratio and CNR may be a much better indicator of need than the CNR often used alone.
>> We are interested in identification of high P:N ratio areas. Assuming (which is plausible in our setting) that the time from diagnosis to notification and initiation of effective treatment is relatively short [12], then if you target neighbourhoods where prevalence is high and TB notifications are also high, the overall TB epidemic is unlikely to be adequately controlled [13]. Given this, the contribution of our approach is to identify areas that are likely to have a high underdiagnosis. Targeting areas with high underdiagnosis is a crucial component in controlling the TB epidemic. Since under diagnosis is also associated with social disadvantage, this also benefits marginalised communities in the city [13].

5. Overall this adds valuable evidence to quantify the extent to which geographical access to TB services contributes to underdiagnosis of TB.

>> Thank you for the positive feedback.

Minor comments:
6. Line 160 – straight line distance from the center of neighbourhoods to the nearest TB clinic is a suitable proxy for ease of access, but I would like to suggest a future refinement could be to use tools including but not limited to AccessMod to capture accessibility in more detail.

>> Thank you for this suggestion; this is indeed an excellent idea for future analysis. We do note that in our previous work in this same setting a straight line distance estimate and a road network distance had the same effect on the rates of TB case fatality [14], providing some support for our use of a straight line distance in this analysis.

7. Table 1: suggest adjusting the columns a little, e.g. Mean (sd) and Range to the left and n/N to the right as the units of per 100,000 or % apply to the mean, rather than the numerator.

>> We have adjusted the columns to make it more apparent where the units belong. We have moved Mean(sd) and Range to be the first columns followed by n/N.

Below is the modified Table1 in the manuscript:

**Table 1: Neighbourhood-level summary data for the 72 neighbourhoods**

| **Characteristic** | **Mean (sd)** | **Range** | **n** | **N** |
| --- | --- | --- | --- | --- |
| 2015 bacteriologically-confirmed adult TB notification rate (per 100,000) | 131 (77) | 0—383 | 479 | 371,834 |
| 2016 bacteriologically-confirmed adult TB notification rate (per 100,000) | 137 (72) | 32—337 | 539 | 403,952 |
| 2017 bacteriologically-confirmed adult TB notification rate (per 100,000) | 120 (60) | 30—307 | 519 | 438,847 |
| 2018 bacteriologically-confirmed adult TB notification rate (per 100,000) | 60 (42) | 0—182 | 283 | 476,763 |
| 2019 bacteriologically-confirmed adult TB notification rate (per 100,000) | 52 (32) | 0—161 | 258 | 517,957 |
| 2019 adult bacteriologically-confirmed TB prevalence rate  (per 100,000) | 215 (335) | 0—1,415 | 29 | 13,490 |
| 2015 adult TB notification rate^†^  (per 100,000) | 242 (119) | 23—638 | 884 | 371,834 |
| 2016 adult TB notification rate^†^  (per 100,000) | 281 (132) | 49—647 | 1,106 | 403,952 |
| 2017 adult TB notification rate^†^  (per 100,000) | 265 (106) | 44.7—477 | 1,157 | 438,847 |
| 2018 adult TB notification rate^†^  (per 100,000) | 138 (71) | 14—312 | 642 | 476,763 |
| 2019 adult TB notification rate^†^  (per 100,000) | 168 (72) | 31—390 | 849 | 517,957 |
| Percentage of adults (≥15y) (%) | 60.90 (3.00) | 54.80—70.60 | 371,834 | 612,635 |
| Percentage of male adults (%) | 51.54 (1.55) | 46.89—55.23 | 191,855 | 371,834 |
| Household head without primary education (%) | 16.90 (6.10) | 4.30—32.40 | 2,700 | 15,897 |
| Distance to TB clinic (km) | 1.73 (0.88) | 0.36—3.66 | NA | NA |
| HIV prevalence (%) | 13.80 (4.32) | 4.21—27.44 | 1631 | 11705 |

8. Suggest some minor rearrangement of the paragraphs on lines 287 – 291 and lines 298 - 301 as there is some repetition between these paragraphs.

>> Thanks, we have reworded the text.

We have reworded the first part of the paragraph and deleted the repeated second part, find quoted text from the manuscript line 307 to line 311

“During 2015-19 there were a total of 2,078 adults aged 18 years or older with bacteriologically -confirmed TB registered for treatment from the study neighbourhoods. CNR’s declined during this period; neighbourhood mean adult bacteriologically-confirmed TB CNR’s from 2015 to 2019 were 131 (range: 0-383, sd: 77), 134 (range: 32-328, sd:70), 114 (range: 28-291, sd:57), 56 (range: 0-167, sd:38) and 46 (0-144, sd:29) per 1000,000 adults Table 1.”

9. Line 385: missing ‘on’ between solely and notification rates.

>> Thanks, corrected.

10. Line 388: With a comprehensive prevalence survey, underdiagnosis becomes readily apparent. I think perhaps the greatest strength of this analysis is that having identified risk factors for underdiagnosis, recent prevalence surveys (while always extremely helpful) may become less necessary.

>> Yes, that is correct. Thank you.

11. Line 401-402: This implies that prevalence survey data is not currently used at all in prioritizing community-based interventions and may need some slight revision.

>> Reworded to the quoted text below.

We have modified the text in the manuscript between line 418 to line 421:

“Our data suggests that the efficiency of selecting communities for community-based interventions, such as those based on poverty and population density, could be improved by incorporating neighbourhood level prevalence survey and notification data which allows a more granular understanding of TB epidemiology [15–17].”

12. Line 460: My interpretation would be that this method is useful for identifying neighbourhoods which are likely to have higher rates of underdiagnosis based on readily available public data, rather than identifying exact neighbourhoods.

>> Yes, that is correct. Also addressed in response to Reviewer #1, Query 27.

**Reviewer #**3: The manuscript uses Bayesian multilevel models to estimate prevalence-to-notification ratios by combining data from registered tuberculosis (TB) patients during 2015-2019 with a survey in 2019-2020.

The paper is well written and I want to commend the authors on addressing the important issue of underreporting of TB cases, but I think that there are some important issues that have to be addressed.

>> Thank you very much.

Major comments:

1. The link to the GitHub repository is not working anymore.

>> The GitHub repository link was previously private. We have now switched it to public. You can now access it. https://github.com/mcewenkhundi/TBhotspotclustersplos

2. You use two independent models: a Poisson model for the confirmed TB case notification rates and a zero-inflated Poisson model for the prevalence data and then you calculate posterior P:N rate ratios by dividing the posterior prevalence rates by the posterior notification rates. Have you considered using a hierarchical model by fitting both models in a common framework? By calculating the P:N ratios on the posterior samples, you can still propagate your uncertainty when using two independent models, but performing the model fitting in two disjoint steps seems somewhat incoherent to me.

>> We used two different, independent datasets, one recording notifications and the other prevalence. Modelling them separately was more technically feasible, allowed greater control in exploring covariates and model structure/variants, and thank you very much for this great idea for future methods research.

We have added text to the manuscript in the Methods section to clarify this point line 188 to line 189:

“Since the notification and prevalence data were from two different and independent datasets they were modelled separately to allow greater control in exploring covariates. The bacteriologically-confirmed case notification data were modelled using a Poisson response distribution (Equation 1), and the prevalence data were modelled using a zero-inflated-Poisson distribution (Equation 2) to account for overdispersion and excess neighbourhoods with zero prevalent cases. Neighbourhood-level random effects were modelled with a spatial intrinsic conditional autoregressive (ICAR) term (S1 and S2 Equations) or a random intercept term (Equation 1 and 2), but not with both random terms in the same model. Possible combination of neighbourhood-level variables were derived for the TB case notification and prevalence data.”

3. Could you explain the rationale of equation 3 in more detail? Why do you only include the fixed and random intercept parameters for both models when calculating posterior prevalence rates and posterior notification rates? In particular, it would make more sense to me to use the posterior notification rates for 2019 since your survey was performed in 2019. Isn’t there a risk that the overall decline in bacteriologically-confirmed TB cases biases your results if you only take the intercept parameters to calculate posterior notification rates? Or did you define the covariates in a way that 2019 is the reference category?

>> Yes 2019 was the reference category. I have added 2019 as a reference category in table 2 in the manuscript and we have also edited equation 1 to make this clearer (refer to Reviewer1 query 4).

|  | **Adult bacteriologically-confirmed TB notification model** | | **Adult bacteriologically -confirmed TB prevalence model** | |
| --- | --- | --- | --- | --- |
| ***Fixed effects* Parameters** | **Mean rate ratio** | **95% CrI** | **Mean rate ratio** | **95% CrI** |
| Percentage of adult residents (≥15y)^a^ | 0.96 | (0.93, 1.00) | 0.94 | (0.80, 1.10) |
| Distance to nearest TB clinic (km)^a^ | 0.78 | (0.69, 0.88) |  |  |
| Percentage of household heads that did not complete primary school^a^ | 0.98 | (0.96, 0.99) |  |  |
| Year: 2019 | Reference |  |  |  |
| Year: 2015 | 2.89 | (2.48, 3.37) |  |  |
| Year: 2016 | 2.91 | (2.51, 3.38) |  |  |
| Year: 2017 | 2.51 | (2.16, 2.92) |  |  |
| Year: 2018 | 1.23 | (1.03, 1.45) |  |  |
| Intercept | 50.88*10^-5^ | (42.99*10^-5^, 60.00*10^-5^) | 232.08*10^-5^ | (132.05*10^-5^, 404.96*10^-5^) |
| Zero inflation intercept |  |  | 0.18 | (0.01, 0.46) |
| **Random effects SD: cluster** | 0.31 | (0.24, 0.39) | 0.33 | (0.01, 0.90) |
| km kilometre; sd standard deviation; Crl Credible interval.  ^a^Percentage of adults was centred by subtracting by its mean (60.90 %), Distance to nearest TB clinic (km) was centred by subtracting by 1km, Percentage of household head that did not complete primary school was centred by subtracting by its mean (16.90 %). | | | | |

4. Did you perform posterior predictive checks to assess model fit for the two models? In MacPherson (2019, BMC Medicine), you plotted predicted and observed TB cases in Additional File 4. I think that such a comparison would also be very helpful to assess the model fit for the models you use here.

>> We have added this in the supplementary material section.

We have done this separately for the prevalence model and the notification model; observed vs fitted (95% Crls) plot. S6 and S7 Figs.

5. It seems as if you considered other covariates in MacPherson (2019, BMC Medicine) for the Poisson model for confirmed TB case notification rates (for instance percentage of population living on less than 2 US dollars) and you used a Besag-York-Mollié prior for the spatial conditional autoregressive structure in TB CNRs. Can you explain why you made these changes in the modelling of TB case notification rates?

>> We explored using the Besag-York-Mollié prior but found we did not have enough information to support the cluster level random effects and the spatial random effects in the prevalence model. While in the supplemental analyses (in the Supplemental Material) we did use the ICAR prior, for the analyses reported in the paper we used only cluster random intercepts. This gave a better description of the data because the spatial proximity of clusters with very different historical levels of intervention caused the ICAR model to over-smooth the data.

The variables that we used in this analysis were informed by the MacPherson (2019 BMC Medicine) paper [1] but we refined our analysis strategy based upon a need to ensure models were robust to the small number of prevalent cases, and data that is easy to collect by National TB Programmes. In particular, this informed our choice to use the household head completion of primary education as a proxy of poverty. (See further discussion of model selection in response to Review#1, query 8 and Review#1 query 10.)

We have modified text to the discussion section to clarify model selection line 446 to line 450:

“We considered modelling neighbourhood random effects with the spatial ICAR term (S1 and S2 Equations and S3 and S4 Tables), but models with random intercept terms gave a better description of the data because the spatial proximity of neighbourhoods with very different historical levels of interventions caused the ICAR model to over-smooth the data (S5 and S6) Tables. In addition, we did not have enough data to support both random effects in the same model.”

6. Is it reasonable to assume that prevalence to notification ratios can be lower than one, i.e. can over diagnosis of TB cases occur? I am just wondering whether the lower credibility intervals for the Prevalence to notification ratios for the neighborhoods with the lowest quartile in Figure 3 make sense.

>>You are right that it is possible for the PNR to be less than 1 year, for example if detection is on average very rapid. Given what we know of TB in a setting like Blantyre, this is unlikely in practice; but we have not constrained the PNRs to be greater than 1 year.

Below is what we have added to the manuscript to acknowledge this limitation line 457 to line 459:

“The lower bound of the 95% CrI of some of the neighbourhood P:N ratios were less than one (Fig 3), although this represents low statistical power rather than a possibility of “overdiagnosis” of TB.”

**References**

1.	MacPherson P et al. Disparities in access to diagnosis and care in Blantyre, Malawi, identified through enhanced tuberculosis surveillance and spatial analysis. BMC Medicine. 2019;17(1):21.

2.	National Statistical Office of Malawi (NSO). Malawi population census preliminary report [Internet]. Zomba; 2018.

3.	Payongayong E et al. Simple household poverty assessment models for Malawi: proxy means test from the 1997–98 Malawi Integrated Household Survey. Lilongwe, Malawi: National Statistical Office, Government of Malawi, 2006.

4.	Malawi National Statistical Office. Intergrated Household Survey (IHS 3) Report 2010-2011. Zomba; 2012.

5.	Vehtari A, Gelman A, Gabry J. Practical Bayesian model evaluation using leave-one-out cross-validation and WAIC. 2016;

6.	Alba S et al. TB Hackathon: Development and Comparison of Five Models to Predict Subnational Tuberculosis Prevalence in Pakistan. Vol. 7, Tropical Medicine and Infectious Disease. 2022.

7.	MacPherson P et al. Disparities in access to diagnosis and care in Blantyre, Malawi, identified through enhanced tuberculosis surveillance and spatial analysis. BMC medicine. 2019;17(1):21.

8.	Borgdorff MW. New measurable indicator for tuberculosis case detection. Emerging infectious diseases. 2004;10(9):1523–8.

9.	Ku C-C et al. Estimated durations of asymptomatic, symptomatic, and care-seeking phases of tuberculosis disease. medRxiv. 2021;2021.03.17.21253823.

10.	World Health Organisation. Global tuberculosis report 2020 [Internet].

11.	Malawi Ministry of Health. Malawi Population-Based HIV Impact Assessment (MPHIA) 2015-2016: Final Report. Lilongwe, Ministry of Health. October 2018.

12.	Ku C-C et al. Durations of asymptomatic, symptomatic, and care-seeking phases of tuberculosis disease with a Bayesian analysis of prevalence survey and notification data. BMC Medicine 2021 19:1. 2021;19(1):1–13.

13.	Dowdy DW et al. Heterogeneity in tuberculosis transmission and the role of geographic hotspots in propagating epidemics. Proceedings of the National Academy of Sciences. 2012;109(24):9557–62.

14.	Khundi M et al. Clinical, health systems and neighbourhood determinants of tuberculosis case fatality in urban Blantyre, Malawi: a multilevel epidemiological analysis of enhanced surveillance data. Epidemiology & Infection. 2021;149.

15.	World Health Organisation. Module 2: Screening WHO operational handbook on tuberculosis Systematic screening for tuberculosis disease [Internet]. 2021.

16.	Wold Health Organisation. Systematic screening for active tuberculosis. Geneva; 2013.

17.	Shaweno D et al. A novel Bayesian geospatial method for estimating tuberculosis incidence reveals many missed TB cases in Ethiopia. BMC Infectious Diseases. 2017;17(1):662.
