# Supplementary material for: Neighbourhood prevalence-to-notification ratios for adult bacteriologically-confirmed tuberculosis reveals hotspots of underdiagnosis in Blantyre, Malawi
Source: PLoS One. 2022 May 23;17(5):e0268749. doi: 10.1371/journal.pone.0268749 (PMC9126376; doi:10.1371/journal.pone.0268749)
Supplement: Attachment — Submitted filename: Response to Reviewers.docx [file pone.0268749.s021.docx]

**McEwen Khundi**
London School of Hygiene & Tropical Medicine and
Malawi-Liverpool-Wellcome Trust, Blantyre, Malawi

P.O Box 30096

Blantyre

Malawi

Mob: +265 997 615 243
Email: [mcewenkhundi@gmail.com](mailto:peter.macpherson@lstmed.ac.uk)

25/04/2022

Dear Editor, PLOS One

Thank you for the helpful review of our manuscript. Please find below our detailed response to all the queries that we were required to address.

Yours Sincerely,

McEwen Khundi

(On behalf of the Authors)

**Editor Comments to Author:**

1. Thank you for submitting your manuscript to PLOS ONE. After careful consideration, we feel that it has merit but does not fully meet PLOS ONE’s publication criteria as it currently stands. Therefore, we invite you to submit a revised version of the manuscript that addresses the points raised during the review process.

>> Thank you.

2. The reviewers acknowledge the efforts by the authors to address the comments and concerns. However, there are a few remaining issues to be addressed before it can be published.

>> Thank you.

3. If you would like to make changes to your financial disclosure, please include your updated statement in your cover letter. Guidelines for resubmitting your figure files are available below the reviewer comments at the end of this letter.

>> We are not changing our financial statement.

**Journal Requirements:**

1. Please review your reference list to ensure that it is complete and correct. If you have cited papers that have been retracted, please include the rationale for doing so in the manuscript text, or remove these references and replace them with relevant current references. Any changes to the reference list should be mentioned in the rebuttal letter that accompanies your revised manuscript. If you need to cite a retracted article, indicate the article’s retracted status in the References list and also include a citation and full reference for the retraction notice.

>> No paper has been retracted in my reference list.

2. While revising your submission, please upload your figure files to the Preflight Analysis and Conversion Engine (PACE) digital diagnostic tool, https://pacev2.apexcovantage.com/. PACE helps ensure that figures meet PLOS requirements. To use PACE, you must first register as a user. Registration is free. Then, login and navigate to the UPLOAD tab, where you will find detailed instructions on how to use the tool. If you encounter any issues or have any questions when using PACE, please email PLOS at figures@plos.org. Please note that Supporting Information files do not need this step.

>> Thank you. We used the link to produce PACE PLOS corrected images.

**Review Comments to the Author**
**Reviewer #1: The authors have done a nice job of addressing my comments, with the exception of the following which warrant further attention:**

1. In the introduction, it is now clearer than before that the authors are proposing to apply their modelling approach to other settings to allow prevalence and PNRs to be characterized with sparser prevalence sampling than would otherwise be required – for“Identification of neighbourhoods with high levels of under-diagnosis without requirement for prevalence surveys”. But this does not come through in the abstract, where it appears they are concluding that [prevalence] “surveys” should be conducted in every city. Please reword the abstract conclusion.

>> Thank you. We have removed the text that suggested that prevalence surveys should be conducted in every city. We have instead added the text below to the Conclusion section of the Abstract (line 41 to line 44).

“Here we have demonstrated a method for the identification of neighbourhoods with high levels of under-diagnosis of TB without the requirement for a prevalence survey; this is important since prevalence surveys are expensive and logistically challenging.”

2. The color variation in Fig 2 remains difficult to see, making the figure unhelpful. Most neighborhoods are in the mid-low range, and the ones that are high (yellow) in early years are very small. Consider shifting the scale (so that there’s more color variation in the low range of 0-200 notifications/100k/year, and in the middle range of 500-1000/100k for prevalence) or using a scale with more color variation (e.g. rainbow).

>> Thank you for this suggestion. We have changed Fig2, and now use the rainbow palette from the viridis R package (rainbow palettethat has been improved for colour blindness “turbo” <https://cran.r-project.org/web/packages/viridis/vignettes/intro-to-viridis.html>)

We have also changed the scale limits for CNR for 2018 to 2019 from 0 to 400 to (0 to 200).

Here is the new Fig2

The previous Fig2 for reference

3. I agree with Reviewer 2 that the exclusive emphasis on high P:N ratios is potentially misleading, and that it would be helpful to include discussion of the fact that CNRs and PNRs should ideally be considered together (rather than looking for high PNRs alone) when prioritizing areas for TB interventions such as active case finding.

>> Thank you. In this study we were predominantly interested in identifying high P:N ratio areas, indicative of under-diagnosis of TB. The reason for this is (i) it is plausible to assume in our setting that the time from diagnosis to notification and initiation of effective treatment is relatively short [1], which in turn implies (ii) if programmes only target neighbourhoods where prevalence is high and TB notifications are also high, the overall TB epidemic is unlikely to be adequately controlled [2].

Nevertheless, we agree with you that it is important to interpret P:N ratios alongside CNRs and PNRs, in order to obtain a full picture of disease activity, and to maximise the effectiveness of control strategies.

We have added the text below in the discussion section to acknowledge this point, lines 479 to line 480:

“The P:N ratios should be interpreted alongside the CNRs to obtain the full nature of the epidemic i.e., Fig 3.”

**Reviewer #2: (No Response)**

**Reviewer #3: Thank you for addressing my comments.**

1. The posterior predictions shown in Figure S6 and S7 actually show that the notification model and in particular the prevalence model fit the data rather poorly. In Figure S6 it seems as if for more than one third (26 out of the 72) of the neighbourhoods the 95% posterior predictive intervals do not include the observed case notification rate. For the prevalence model, the situation seems even worse because only 8 out of the 72 prevalence rates are included in the 95% posterior predictive intervals. This latter point is clearly linked to the zero-inflation part of the zero-inflated Poisson model, but it almost looks as if the zero-inflation hadn't worked properly because you would expect such poor predictions if you had a zero-inflated Poisson process and you fitted a regular Poisson model. Can the authors comment on this point? Correct me if I am naive, but I wouldn't trust models for which the posterior predictive distributions are so far off the observed data and I think that it would be important to acknowledge this in the manuscript and to suggest reasons for this discrepancy.

>> Thank you.

Figures S6 and S7 show the (posterior) predicted means and their 95% CIs for each of the areas. These are not prediction intervals for a new observation. Therefore, even when the data are actually generated from the true model, these will not include the observed data 95% of the time.

However, the question of goodness of fit of the model is very important, but not entirely straightforward in this situation, in part because of the small numbers involved (particularly in the prevalence data).

The section below has been added to the supplementary section (S3 Equation, model goodness of fit assessment) just below Figures S6 and S7 in the manuscript.

We now present a chi-square-statistic goodness of fit statistic for the models as an approximate guidance to the goodness of fit of the models to the data.

Let $Y_{i}$ be the observed number of cases and $\hat{\mu_{i}}$ the model predicated cases in neighbourhood *i* = 1, 2, 3, …72

$$\chi^{2}=\sum_{i} \frac{{(Y_{i}- \hat{\mu}_{i})}^{2}}{\hat{\mu}_{i}}$$

The value of chi-square was calculated separately for TB case notifications and also for prevalent TB cases. We also calculated the approximate degrees of freedom of each as

72 – number of parameters in each model.

The calculated chi-squared statistic of the case notifications was 73.06, while the degree of freedom was 64, giving an approximate p-value of 0.21

**Similarly,** the calculated chi-squared statistic of the prevalent cases was 56.81, while the degree of freedom was 70, giving an approximate p-value of 0.87.

While, because expectations in the 72 locations are often small, this only provides an approximate measure of goodness of fit, nevertheless taken together with our careful choice of models (e.g. zero-inflated Poisson for the prevalence) and systematic variable selection approach means are confident our models describe the data sufficiently well to justify their application.

We have changed the text in the discussion on lines 467 and 469:

“More details on the goodness of fit of the models have been provided in the supplementary section (S3 Equation), which suggests that our models sufficiently describe the data well. “

**S6 Fig. Observed versus predicted mean CNRs (95% Crls). Analysis based on microbiologically-confirmed TB as in the primary analysis.** Crl Credible interval.

**S7 Fig. Observed versus** **predicted mean prevalence rates (95% Crls). Analysis based on TB prevalence as in the primary analysis.** Crl Credible interval.

**References**

1.	Ku C-C et al. Durations of asymptomatic, symptomatic, and care-seeking phases of tuberculosis disease with a Bayesian analysis of prevalence survey and notification data. BMC Medicine 2021 19:1. 2021;19(1):1–13.

2.	Dowdy DW et al. Heterogeneity in tuberculosis transmission and the role of geographic hotspots in propagating epidemics. Proceedings of the National Academy of Sciences. 2012;109(24):9557–62.

3.	World Health Organisation. Module 2: Screening WHO operational handbook on tuberculosis Systematic screening for tuberculosis disease [Internet]. 2021.
